# A New Venue of TNF Targeting

**DOI:** 10.3390/ijms19051442

**Published:** 2018-05-11

**Authors:** Sophie Steeland, Claude Libert, Roosmarijn E. Vandenbroucke

**Affiliations:** 1 Barriers in Inflammation, VIB Center for Inflammation Research, Ghent, Department of Biomedical Molecular Biology, Ghent University, 9052 Ghent, Belgium; Sophie.Steeland@irc.VIB-ugent.be; 2 Mouse Genetics in Inflammation, VIB Center for Inflammation Research, Ghent, Department of Biomedical Molecular Biology, Ghent University, 9052 Ghent, Belgium; Claude.Libert@irc.VIB-ugent.be

**Keywords:** tumor necrosis factor (TNF), TNF receptor (TNFR), biologicals

## Abstract

The first Food and Drug Administration-(FDA)-approved drugs were small, chemically-manufactured and highly active molecules with possible off-target effects, followed by protein-based medicines such as antibodies. Conventional antibodies bind a specific protein and are becoming increasingly important in the therapeutic landscape. A very prominent class of biologicals are the anti-tumor necrosis factor (TNF) drugs that are applied in several inflammatory diseases that are characterized by dysregulated TNF levels. Marketing of TNF inhibitors revolutionized the treatment of diseases such as Crohn’s disease. However, these inhibitors also have undesired effects, some of them directly associated with the inherent nature of this drug class, whereas others are linked with their mechanism of action, being pan-TNF inhibition. The effects of TNF can diverge at the level of TNF format or receptor, and we discuss the consequences of this in sepsis, autoimmunity and neurodegeneration. Recently, researchers tried to design drugs with reduced side effects. These include molecules with more specificity targeting one specific TNF format or receptor, or that neutralize TNF in specific cells. Alternatively, TNF-directed biologicals without the typical antibody structure are manufactured. Here, we review the complications related to the use of conventional TNF inhibitors, together with the anti-TNF alternatives and the benefits of selective approaches in different diseases.

## 1. Introduction

Since the discovery and identification of tumor necrosis factor (TNF) in the mid 1980s [1], novel techniques allowed the isolation and cloning of the TNF gene for further characterization and TNF became the subject in a lot of studies. Originally, TNF was identified as a factor that necrotizes certain tumors [2,3] and recombinant TNF was useful to discover the biological functions of TNF. As a tumor-necrotizing agent, TNF’s toxicity in animal models was apparently acceptable, thus TNF was quickly launched into clinical trials. Eighteen monotherapy phase I and 10 phase II clinical trials were performed using recombinant human TNF (hTNF) therapy as anti-cancer agent, but none of them was successful as systemic TNF treatment was found to cause dose-dependent toxicities such as fever, hypotension and tachycardia [4,5,6]. Based on these and other studies, it became clear that TNF is a pleiotropic cytokine with major roles in physiology and pathology, amongst others by causing necrotic and apoptotic cell death, cellular regulation, differentiation, inflammation and the regulation of immune cells, and tumorigenesis [7]. The executive functions of TNF exceed multiple disciplines as TNF is important in homeostatic processes as well as in pathological situations ranging from inflammation, neurodegenerative diseases and infections.

Today, 19 members of the TNF superfamily have been identified, based on sequence similarity with TNF. In addition, 29 interacting receptors and several molecules interacting with the cytoplasmic domain of these receptors are recognized [1,7]. All members of the TNF receptor (TNFR) family contain one to six cysteine-rich repeats in their extracellular domains, typically each with three cysteine bridges within their structure [8]. The receptors can be classified in two subgroups: the death domain (DD)-containing receptors and the tumor necrosis factor receptor-associated factor (TRAF)-interacting receptors [9].

## 2. Biology

TNF is expressed as a 26 kDa transmembrane protein (tmTNF) which can be shed by the metalloproteinase TNF-α-converting enzyme (TACE) or disintegrin and metalloproteinase 17 ADAM17 to release the homotrimeric soluble TNF form (sTNF, monomeric 17 kDa) [10]. TNF is produced by a variety of cell types, such as monocytes and macrophages, T cells, natural killer (NK) cells, neutrophils, and microglia as well as by non-immune cells such as neuronal cells or keratinocytes. Both tmTNF as well as sTNF are biologically active, and the balance between these two forms is influenced by the cell type and its activation status, TACE activity and the expression of the endogenous TACE inhibitor, tissue inhibitor of metalloproteinase (TIMP)-3 [11,12]. TNF binds two homotrimeric transmembrane receptors: the 55 kDa TNF receptor 1 (TNFR1 or CD120a), encoded by the *TNFRSF1A* gene and the 75 kDa TNF receptor 2 (TNFR2 or CD120b), encoded by *TNFRSF1B* [9]. Interestingly, instead of only being a ligand, tmTNF can also act as a receptor because tmTNF-bearing cells show biological activity via reverse signaling when activated by mainly TNFR2. However, the biological functions elicited by this “outside-to-inside signaling” have not been completely elucidated [13]. TNFR1 is constitutively and ubiquitously expressed on a broad variety of cells, whereas expression of TNFR2 is inducible and tightly regulated. TNFR2 expression is more restricted and can be typically found on endothelial, immune (including microglia) and neuronal cells [9]. Recently, TNFR2 has also been found to be expressed on tumor cells and has been suggested to function as a tumor oncogene [14,15]. The extracellular domains of the two receptors are conserved and consist of a pre-ligand assembly domain (PLAD) and a ligand-binding domain, which is composed of four cysteine-rich domains and a TACE substrate domain. The PLAD stabilizes the receptors in absence of ligand as homophilic dimers. PLAD-mediated receptor preassembly is necessary for TNF/TNFR signaling and deletion of PLAD completely abrogates ligand binding and signaling [16]. In contrast to their extracellular domains, their intracellular domains are unrelated, explaining the initiation of different signaling cascades [17]. TNFR1 is a DD-containing receptor allowing protein–protein interactions, while TNFR2 does not have such a DD [18,19]. Successful signaling via TNF requires receptor preassembly as trimers prior to ligand binding. Preassembly occurs through the intracellular cytoplasmic tail of the receptors. The DD can recruit two adaptor DD-containing proteins, namely TNFR1-associated death domain (TRADD) or Fas-associated death domain (FADD), whereupon the apoptotic pathway is activated and the caspase cascade is engaged [20]. Importantly, in addition to other ligands such as Fas and TRIAL, TNF via TNFR1 can activate a caspase-independent pro-inflammatory cell death, called necroptosis [21,22,23]. This is a relatively novel programmed necrosis-like inflammatory process.

Upon TNFR2 activation, this receptor recruits TRAF2 and other TRAF2-associated proteins, as well as interacts with other signaling proteins that act independently of TRAF2. Whereas TNFR1 is linked with pro-inflammatory and apoptotic effects, TNFR2 has been associated with a variety of immune regulatory and anti-inflammatory functions [20]. Importantly, a complex interplay between TNFR1 and TNFR2 has been described, and additive, synergistic as well as antagonistic effects have been demonstrated [9].

TNFR1 is activated by either sTNF as well as tmTNF, while TNFR2 can only be activated by tmTNF. Hence, the role of TNFR2 is thought to be underestimated [24]. The membrane-bound forms of both receptors are also a substrate for proteolytical cleavage by TACE, yielding soluble receptor fragments e.g., soluble TNF receptor (sTNFR) [25]. This process is an important self-regulatory mechanism to prevent exaggerated damage and may contribute to the regulation of cellular TNF responsiveness [25]. Increased ectodomain shedding has three consequences: (1) On the one hand, the shed receptors can neutralize the bioactivity of circulating TNF by sequestering it. Hence, sTNFR will act as an intrinsic TNF inhibitor. (2) On the other hand, the process will decrease the number of signaling-competent receptors on the cell surface and cause transient TNF desensitization [26]. Accordingly, mice expressing non-sheddable TNFR1 spontaneously develop liver pathology and autoimmunity, pointing towards the pivotal role of TNFR1 shedding to regulate TNF activity in vivo [27]. The importance of this system is also highlighted in the disease condition of TRAPS (TNF receptor-associated periodic syndrome), an autosomal dominantly inherited disease characterized by unprovoked, often prolonged, attacks of fever and inflammation in multiple organs caused by a mutation in *TNFSFR1A* [28]. (3) Alternatively, sTNFR1 can form a stable complex with sTNF which can act as a sink in which the circulating TNF levels will be preserved.

## 3. TNF in Health and Disease

TNF is a pleiotropic cytokine and a master regulator of the immune system, and most cells show at least some TNF responsiveness. Studies revealed two opposite functions of TNF in host defense. At low levels, TNF has beneficial homeostatic functions, such as for host defense mechanisms against for example intracellular pathogens, particularly mycobacteria such as *Mycobacterium tuberculosis* [29]. However, at high concentrations, TNF can be deleterious and promotes inflammation and organ injury. In disease states, TNF is predominantly secreted by macrophages and monocytes both systemically and locally in the affected tissues, but also many other cells can produce TNF under certain circumstances [30].

### 3.1. Homeostatic Functions of TNF in Immunity

The use of transgenic mice greatly enhanced our knowledge about the different functions of TNF. TNF deficient mice revealed that TNF has prominent homeostatic functions in addition to its pro-inflammatory roles, and is vital for an optimal functioning of our immune system needed for host defense. Indeed, it has been shown that TNF is required to develop splenic B cell follicles, for the organization of the secondary lymphoid tissue architecture, and for germinal center (GC) and follicular dendritic cell (FDC) formation. Additionally, TNF is indispensable to fight pathogens and prevent further pathogen spreading by the formation and maintenance of granulomas, which are organized accumulations of infected macrophages and lymphocytes [31,32]. Likewise, TNF is highly needed for the resolution of inflammation and to promote tissue repair illustrated by its need for neuronal remyelination, cardiac remodeling and cartilage regeneration. The homeostatic potential of TNF has been proposed to be mainly mediated by TNF/TNFR2 signaling [33,34,35].

As mentioned earlier, the indispensable role of TNF for host defense against (intracellular) bacteria, viruses and fungi is well documented. Indeed, alone, together, or in synergy with interferons (IFNs), TNF is the most potent mediator in this process and it needs both TNF receptors for an efficient defense. Via the induction of chemokines and adhesion molecules [36], TNF is indirectly involved in the recruitment of inflammatory innate immune cells such as neutrophils, monocytes, NK cells and the so-called antigen presenting cells (APCs): immature tissue resident macrophages and dendritic cells (DCs). TNF enhances the pathogen-directed cytotoxicity of the innate immune cells and acts as a stimulatory agent for phagocytes [37]. In addition, TNF-induced NF-κB is required to convert immature tissue resident DCs into functionally mature effector cells. The latter will then stimulate naive T cells in nearby draining lymph nodes to initiate antigen-specific T and B cell responses [38]. For example, TNF is essential for a normal host response to *Mycobacterium bovis* and, more specifically, TNF derived from hematopoietic cells rather than from stromal origin [39]. To control *M. tuberculosis*, it has been shown that myeloid and T cells are the primary sources of TNF: myeloid cell-derived TNF is implicated in early immune responses, whereas T cell-derived TNF is essential to sustain protection during chronic infections [40].

Most of the pro-inflammatory functions are mediated by TNFR1, as mice deficient for TNFR1 are highly susceptible to *Listeria monocytogenis* and *M. tuberculosis* infection, and TNFR1 is also required for the anti-viral response via induction of apoptotic cell death [38,41]. Moreover, loss-of-function due to TNF knockout is highly mimicked by TNFR1 deficiency [42]. The role of TNF/TNFR1 signaling in *M. tuberculosis* infection relates to its contribution in granuloma formation that is needed to control the infection. Additionally, it may relate to macrophage activation to produce reactive nitrogen species to destroy intracellular bacteria [43]. Interestingly, mice that express a non-cleavable variant of TNF were partially protected against *M. tuberculosis*, *L. monocytogenes* and *M. bovis*, indicating that tmTNF signaling, which predominantly occurs via TNFR2, is needed for this protection [44]. In addition, mice that express non-cleavable TNFR1 were more resistant to *L. monocytogenes* infections, suggesting that impaired TNFR1 shedding enhances the antibacterial host defense [27]. Additionally, a recent study indicates that TNFR1 on myeloid and not on T cells is crucial to control *M. tuberculosis* infection [45].

### 3.2. Systemic Inflammation

#### 3.2.1. TNF: The Master Regulator of Inflammation

**Figure 1:**
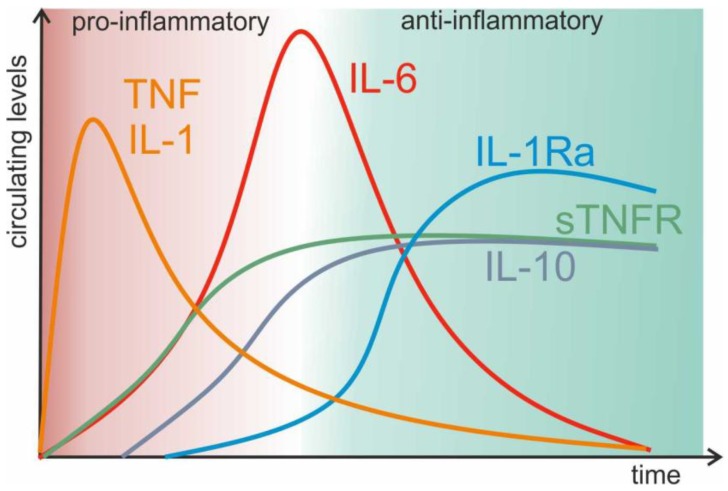
Cytokine kinetics in sepsis. Tumor necrosis factor (TNF) and interleukin (IL-)1 are the first cytokines to be released in sepsis and promote the secretion of IL-6. Together, these cytokines are the orchestrators during the pro-inflammatory phase in sepsis. After some time, compensation mechanisms arise to dampen the pro-inflammatory response such as IL-10, IL-1 receptor antagonist (IL-1Ra) and soluble TNF receptor (sTNFR). Figure adapted from [63].

Sepsis is a very complex syndrome that is caused by a dysregulated host response to infection. It is characterized by sustained excessive inflammation and immune suppression, and many studies have shown that TNF is the master mediator of the inflammatory response seen in sepsis and shock, the life-threatening condition caused by circulatory and/or metabolic abnormalities. Indeed, TNF is released from macrophages into the systemic circulation as early as 30 min after an inciting event such as an intraperitoneal (ip) lipopolysaccharide (LPS) injection, with peak concentrations observed after 60–90 min [46]. This primes the activation of other inflammatory mediators such as interleukin (IL-)1. Depending on where TNF is produced and on which cell it acts, TNF not only potently promotes the release of the secondary mediator IL-6, but also drives its own production. The excessive production of pro-inflammatory mediators is followed by a wave of counteracting and anti-inflammatory mediators such as soluble TNFR that sequesters its bioactive ligand [47] (Figure 1). During bacterial infections, these cytokines are the main drivers and are the central mediators of the induced shock after either Gram-positive or Gram-negative bacteremia [38,48]. Thus, dysregulation of the TNF production due to an overreaction of the host may have unforeseeable consequences. In animals, exogenous TNF administration leads to a syndrome that is indistinguishable from septic shock and infusion of TNF into humans results in systemic inflammatory response syndrome (SIRS) [49]. Consequently, sustained elevated endogenous TNF levels can lead to a SIRS, which may evolve to death due to multiple organ failure (MOF). Several studies demonstrate that TNF serum levels in sepsis patients are elevated and associated with mortality [50,51] and they are used as effective markers in the diagnosis of neonatal sepsis [52]. These observations led to the rationale to therapeutically neutralize circulating TNF in septic patients. However, numerous clinical trials failed to demonstrate clear statistical benefits [53,54,55,56,57,58,59]. As sepsis involves the presence of a pathogen, the inability to control the infection when TNF signaling is abrogated might account for the failure seen in these clinical trials. Indeed, in sterile sepsis models (i.e., LPS-induced shock), anti-TNF treated mice show some degree of protection but this is not recapitulated in real infection models such as cecal ligation and puncture (CLP) [60,61]. However, a meta-analysis suggests that immunotherapy with monoclonal antibodies (mAbs) against TNF does reduce the overall mortality in severe septic patients when the drugs is administered before shock. Furthermore, it may improve survival in patients with shock or with high IL6 levels, and thus requires patient stratification [62].

#### 3.2.2. Differential Roles for TNFR1 and TNFR2 in Sepsis

In sepsis, a differential role for TNFR1 and TNFR2 has been uncovered by using transgenic mice in several experimental models, although their exact contributions remain debatable. Mice deficient for TNFR1 were protected against death when they were subjected to endotoxemia, i.e., the injection of a lethal dose of LPS [64,65], whereas TNFR2 knockout (KO) mice were as sensitive as wild type (WT) mice [66]. At the level of the gut, Williams et al. reported that TNFR1 is essential for LPS-induced gut injury by mediating apoptosis of intestinal epithelial cells (IECs) [67], and also in the TNF-induced lethal shock model, the complete survival benefit in TNFR1 KO mice was attributed to the avoidance of TNF-induced gut permeability [41,68,69]. Additionally, the blood–cerebrospinal fluid (CSF) barrier permeability was also less comprised in endotoxic TNFR1 KO mice [70]. This is an important observation, as preservation of the integrity of the brain barriers might overcome the occurrence of sepsis-associated encephalopathy (SAE) [71]. This devastating complication of sepsis is associated with early death in sepsis patients [72]. It has been suggested that TNFR1 is a critical mediator in the onset of SAE because of its stimulating effects on aquaporin-4 and concomitant increase in water content in the brain [73]. Interestingly, TNFR1^−/−^ mice also experienced less sepsis-induced memory deficits [74]. However, despite of these interesting observations, TNFR1 KO mice were not protected against very high LPS doses [42,66], which was also confirmed in our lab [70]. The previous models are sterile models, and observations in CLP, which is considered as the golden standard for human polymicrobial sepsis [75], or in colon ascendens stent peritonitis (CASP) left the scientific community with contradictory results regarding the different roles of TNFR1 and TNFR2 in real sepsis models. Some groups, including ours, found that TNFR1 deficient mice were as sensitive as WT mice in CASP or CLP [70,76,77], whereas others reported that TNFR1 KO mice had prolonged survival in CLP and that TNFR2 KO mice were more sensitive than WT mice [78]. When polymicrobial sepsis was initiated by ip injection of cecal microflora, the levels of TNF were severely elevated and mice deficient for TNFR1 or both TNFRs survived the induced sepsis [61]. Collectively, these studies fail to provide a clear consensus about the exact contribution of each receptor. These data also suggest that lethality does not only depend on TNF, but also other inflammatory mediators contribute to the LPS-induced lethality for instance IL-1β or matrix metalloproteinases (MMPs) [79]. Indeed, former research of the lab reported an important interplay between TNFR1 signaling and MMP8 in sepsis [70]. We found a link between these two mediators in sepsis patients and mice deficient for the both genes were significantly more protected against very lethal endotoxemia and CLP-induced peritonitis than WT or single KO mice. Hence, our research group created a bivalent Camelid-derived heavy-chain only Ab, namely Nanobody (Nb) 70-alb-14, that simultaneously antagonizes TNFR1 and MMP8. Proof-of-concept was provided as treatment with this Nb reduced lethality against CLP [70].

### 3.3. Autoimmunity

#### 3.3.1. Implications of TNF Signaling

Persistently elevated levels of TNF are evident in chronic inflammatory disorders, e.g., rheumatoid arthritis (RA), inflammatory bowel disease (IBD), ankylosing spondylitis (AS) and psoriasis. TNF regulates differentiation, proliferation and apoptosis of several non-immune cells such as IECs, keratinocytes or synovial fibroblast [80,81,82]. In IBD, overexpression of TNF may contribute to increased barrier permeability for luminal pathogens [81]. Importantly, TNF can also initiate necroptosis and this is a common feature in the pathology of several inflammatory diseases and autoimmunity such as in Crohn’s disease (CD) [21,22,23]. The pro-inflammatory role of TNF in these autoimmune diseases is supported by the great clinical effects of TNF-antagonists, which are used in these disorders both in humans as well as in animal models of for instance RA [83,84]. Again, genetically modified mice were an invaluable tool to elucidate the pathogenic role of TNF here. Many valuable insights came from transgenic mice that lack the AU-rich element (ARE) in the 3′-untranslated region of the TNF mRNA. TNF^∆ARE^ mice systemically overexpress TNF due to the increased stability of its mRNA. They spontaneously develop erosive, symmetric polyarthritis and IBD. As the clinical symptoms in the mice resemble the clinical and histopathologic features of RA and IBD in patients, they are an ideal model for these diseases [30,85]. Furthermore, other transgenic mice expressing a hTNF transgene were found to develop a TNF-dependent chronic inflammatory polyarthritis resembling human RA [86,87]. However, this does not necessarily mean that all types of RA and IBD in patients start with a dysregulated TNF expression.

Despite the untoward effect of TNF in the development of autoimmunity, it has also been demonstrated that TNF is sometimes needed to inhibit or control autoimmunity [88]. Indeed, TNF neutralization exacerbates acute injury in the dextran sodium sulfate (DSS) colitis mouse model [89] and exogenous TNF administration could alleviate colitis in oxazolone-treated mice and attenuate the disease severity in RA [90,91]. Furthermore, studies demonstrate that chronic treatment with a low TNF dose or local pancreatic expression of TNF in adult non-obese diabetic (NOD) mice, which is a model for diabetes, delayed spontaneous development of type I diabetes in these mice [92,93]. A similar finding was done in NZB/W mice, a model for systemic lupus erythematosus (SLE), in the development of autoimmunity [94]. Additionally, when these lupus-prone mice were crossed into a TNF deficient background, they experienced aggravated disease [95]. In addition to suppressing systemic autoimmunity, TNF can also suppress organ-specific autoimmunity. The importance of this mechanism is further highlighted in MS mouse models. In the absence of TNF, myelin-specific deleterious T cells remain abnormally prolonged self-reactive to myelin, while in normal conditions they become inactivated [96]. Therefore, TNF may be protective against chronic experimental autoimmune encephalomyelitis (EAE) by downregulation or inactivation of the potentially detrimental autoimmune T cell response against myelin antigens [88]. In conclusion, many models suggest an immunosuppressive and immunoregulatory role for TNF, which may segregate at the level of the two different receptors: the classic pro-inflammatory activities of TNF mediated by TNFR1 may account for chronic inflammatory pathology and tissue damage, especially in situations with persistent and maintained TNF overexpression [88], while immunosuppressive activities might be attributed to TNFR2 [97].

#### 3.3.2. Receptor-Dependent Roles for TNF in Autoimmunity

To analyze the contribution of the two TNF receptors, the aforementioned experimental mouse models were applied in a TNFR1 and/or TNFR2 KO background. In the collagen-induced arthritis (CIA) model for RA, TNFR1 KO mice developed the disease at lower incidence and in a milder form than WT mice [83,98]. In contrast, TNF-driven arthritis was severely aggravated in TNFR2 KO mice [35,99]. The importance of TNFR2 signaling in autoimmune diseases is also illustrated by its prominent role in regulatory T cell (T_reg_) functioning [100,101,102]. The most potent T_regs_ express the highest TNFR2 levels and TNF/TNFR2 signaling is required to activate and expand naturally occurring T_regs_ [101,103,104]. Given that T_regs_ are essential for immune tolerance and suppress self-reactive T cells [105], their optimal functioning should be considered in new therapies. In RA, selective inhibition of TNFR1 abrogates inflammation by enabling T_regs_ to suppress IL-17 production, and promotes T_reg_ activity via TNFR2 signaling [106,107]. Likewise, TNFR1 or double TNFR deficient TNF^∆ARE^ mice had a normal histology without any sign of macroscopic illness. In contrast, symptoms were not improved but rather aggravated with more aggressive and destructive arthritis when crossed into the TNFR2 KO background [85]. In addition to these data, mouse and human data in CD point to the importance of the suppressive functions of T_regs_ which are attributed to TNFR2 [108,109]. Indeed, in mice, T_regs_ are critical for maintaining intestinal tolerance to luminal antigens and for preventing intestinal inflammation [110]. Genetic data further strengthen the importance to acknowledge the two receptors separately. Indeed, polymorphisms in (the promoter region of) *TNFSF1B* were associated with increased susceptibility for patients to develop RA, IBD or lupus, suggesting that TNFR2 mutations could lead to increased inflammation due to defective control mechanisms [111,112]. However, the effects of these polymorphisms on the T_reg_ population are not studied and require further examination [113].

### 3.4. The Role of TNF in Neurodegenerative Diseases

The most under-appreciated role of TNF is its role in neurobiology. In the last decades, TNF has been shown to have several important physiological but also pathological functions in the CNS [12]. In the brain, the duality of TNF is nicely demonstrated at several levels. On the one hand, TNF functions as an essential gliotransmitter, secreted by neurons and glial cells such as microglia and astrocytes. Moreover, TNF regulates synaptic communication between neurons as demonstrated by its involvement in synaptic scaling and plasticity [114], and it orchestrates learning and memory processes via hippocampal neuronal development [115]. Other neurophysiological functions of TNF are listed by Decourt et al. [116]. On the other hand, TNF potentiates excitotoxicity via a microglial and astroglial loop that ultimately results in neuronal death, and inhibition of sTNF ameliorates synaptic dysfunction in aging and improves learning [117]. Conversely, TNF also protects against excitotoxicity via TNFR2 [12,118,119].

Intriguingly, systemic inflammation induces TNF expression in the brain [120] and it has been shown that in mice, TNF can cross the blood–brain barrier (BBB) to reach the brain via a saturable transport system [121]. Microglia which are the tissue-resident macrophages of the CNS are one of the major producers of TNF, participating in numerous pathophysiological conditions in the brain. Indeed, elevated TNF levels are evident in many neurological disorders such as in affected areas in multiple sclerosis (MS, cf. Section 3.4.2), Alzheimer’s disease (AD, cf. Section 3.4.3), Parkinson’s disease (PD, cf. Section 3.4.4), stroke and traumatic brain injury (TBI). Intriguingly, TNF, amongst others, mediates necroptosis which is clearly involved in AD and PD, highlighting one of the possible detrimental effects TNF can exert in these disorders [122]. TNF released in the brain can be both toxic and tropic; however, this is not always clear and depends on the context [12,123,124]. Therefore, unselective targeting of TNF in the brain with therapeutics is not desirable and distinctions should be made based on its function.

#### 3.4.1. Differential Roles of TNFR1 and TNFR2 in Neuronal Health and Disease

In the CNS, microglia secrete TNF and it has been shown that TNF promotes its own release via TNFR1. Hence, it sustains a vicious feedback loop in microglial activation [125]. Additionally, TNFR1 signaling upon TNF interaction could induce the release of glutamate from microglia and astrocytes and also directly potentiates glutamate excitotoxicity through the activation of the glutamate α-amino-3-hydroxy-5-methyl-4-isoxazolepropionic acid (AMPA) receptors [125,126,127]. Conversely, TNFR2 activation protects against glutamate-induced excitotoxicity [119,128]. In aging, it has been suggested that TNF/TNFR1 signaling contributes to cognitive decline through its effects on hippocampal long-term depression (LTD). Indeed, in aged rats, the hippocampal levels of the TNF receptors are changed in favor of TNFR1, and inhibition of the TNF/TNFR1 pathway improves behavioral performances and synaptic functioning [117]. Literature also suggests a direct link between the activation of TNFR1 by TNF and neuronal apoptotic death in neurodegenerative disorders [129,130]. Additionally, neurons lacking TNFR2 are more vulnerable to TNF-induced cell death than WT neurons, as TNFR2 overrides the death signals delivered through TNFR1 [131]. In a disease state, TNF robustly stimulates TNFR1 resulting in an apoptotic signal that overweighs the signals through TNFR2. In this respect, hippocampal neurons from TNFR2^−/−^ mice are more vulnerable to a low TNF dose whereas TNF has little effects in TNFR1^−/−^ hippocampal neurons [130]. During neurogenesis, TNF via TNFR1 negatively impacts the hippocampal neurogenesis but is essential for striatal morphology and injury resolution. Conversely, TNFR2 is required for normal hippocampal neurogenesis and morphology in healthy adults, and for hippocampal healing upon injury [132,133,134,135]. In addition, in the context of neuropathic pain, the induced depression and impaired hippocampal plasticity depend on TNFR1 signaling [136]. During stroke, optimal TNF signaling is pivotal for hippocampal neurogenesis, functioning and repair following ischemic insults [137,138]. Table 1 provides a non-exhaustive list of neurological conditions in which different roles for TNFR1/2 are described, and we will deeper dig into MS, AD and PD.

#### 3.4.2. TNF and Its Receptors in Multiple Sclerosis

MS is characterized by immune cell infiltration and upregulation of pro-inflammatory cytokines and chemokines such as IL-1β, IL-17, IL-22, IFN-γ and TNF in CSF [139,140]. An important role for TNF in MS has been described although its exact role remains inconclusive. TNF and its receptors are found in the serum, CSF and lesions of MS patients and TNF levels in serum and CSF are correlated with disease severity [140,141]. Additionally, mouse studies using the established MS mouse model EAE revealed a pathogenic role for TNF in MS [142,143] and CNS-specific overexpression of TNF leads to spontaneous demyelination [144,145]. It has been suggested that TNF-mediated demyelination depends on cellular contacts between TNF-producing cells such as astrocytes or microglia and oligodendrocytes (OLGs). Indeed, human tmTNF expressed by astrocytes is more effective to kill OLGs than sTNF and astrocyte-specific overexpression of tmTNF triggers inflammation and demyelination and this is mediated via TNFR1 [145,146]. It was shown that T cells and myeloid cells are the critical cellular sources of TNF during EAE as T cell-derived TNF determines the severity in EAE by regulating leukocyte influx into the CNS, whereas myeloid-derived TNF controls the early expression of cytokines and determines the onset of EAE [147]. Evidence of its pathogenic role was further provided by anti-TNF treatment that prevented the initiation of clinical symptoms in EAE and ameliorated progression in established disease in mice [148]. To the contrary, although initiation of EAE in TNF KO mice was delayed, the mice eventually developed EAE that was as severe or even more severe with high mortality and extensive inflammation as compared to WT mice [149,150,151]. Furthermore, anti-TNF treatment increased MRI activity and immune activation in several patients with primary progressive MS (PPMS) [152] and in patients with relapsing-remitting MS (RRMS), a phase II clinical study with a TNF inhibitor (Lenercept, a dimeric TNFR1 fused to the immunoglobulin (Ig)-G1 heavy chain) was discontinued because of unexpected exacerbations of the disease [153]. Strikingly, anti-TNF medication can even sporadically induce demyelinating diseases and neuropathies (cf. Section 4.3.4), and several groups found that TNF expressed in lymphoid organs could dampen the immune response by inhibiting the development of encephalitogenic T cells responses [96,147]. Contrasting results have also been described in OLGs as TNF is involved in oligodendrocyte precursor cell (OPC) proliferation and maturation [33] but also causes OLG cell death both in vitro and in vivo [154,155,156,157]. OLGs located at the edge of active lesions express both TNFRs and this could explain the duality of TNF in MS. These results suggest that TNF is again not only destructive but also has essential roles to maintain immune homeostasis in the CNS environment.

**Figure 2:**
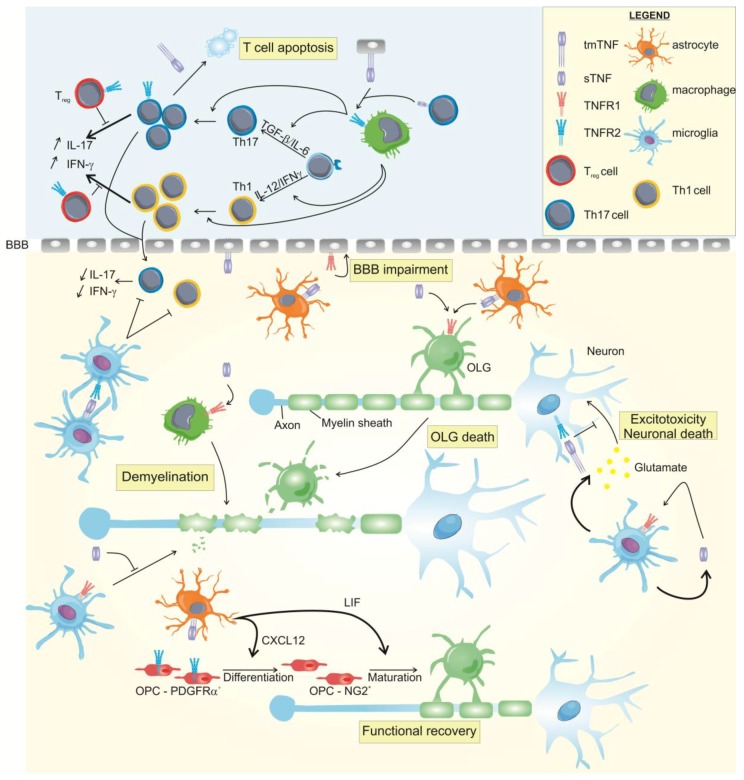
Multiple roles for tumor necrosis factor (TNF) receptor (TNFR) signaling in multiple sclerosis (MS) pathology. In the central nervous system (CNS), TNF is primarily expressed by astrocytes, microglia and neurons and can stimulate its own release via TNFR1. A detrimental role for TNFR1 has been described in the pathology of MS. TNFR1 signaling triggers oligodendrocyte (OLG) death and contributes to primary demyelination via macrophages. Conversely, TNFR2 has protective effects in the CNS, as the interaction with tmTNF on astrocytes stimulates remyelination and neuronal TNFR2 protects against excitotoxicity. In the periphery, TNFR2 induces the development of effector T cells, but in the CNS, microglial-expressed TNFR2 is protective. TNF, possibly via TNFR2, mediates regression of activated myelin-specific T cells. Furthermore, TNFR2 signaling also facilitates the expansion of regulatory T cells (T_regs_) and improves their suppressive capacities against effector T cells. Normal arrows indicate the action of a mediator or the processes that are induced; bold arrows represent mediators produced by a specific cellular subset. T bars represent the inhibition of the indicated pathway.

Interestingly, the *TNFRSF1A* locus has been validated as a MS susceptibility locus [158]. The disease genetic variant leads to the expression of a soluble form of TNFR1 that sequesters TNF and thereby abrogates signaling through TNFR2. This suggests that dysregulation of the TNF/TNFR1-pathway has a role in the onset of MS [159]. TNF/TNFR1 signaling has been shown to be involved in OLG apoptosis, demyelination, and initiation of inflammatory processes (Figure 2 and Table 1). First evidence of the harmfulness of TNFR1 has been delivered as EAE disease development was prevented in double TNFR KO as well as in TNFR1 KO mice. This was contrasted by the outcome of TNFR2 null mice which developed a more severe and chronic disease [160,161]. Interestingly, both TNFR1 and TNFR2 KO mice were able to suppress the anti-myelin reactivity, leading to the idea of redundancy in the immunosuppressive functions of the two receptors. However, again, TNFR1 was found to be responsible for the detrimental signals, while mainly TNFR2 was essential for resolving the inflammation and initiating repair [161]. Additionally, in TNFR1 deficient mice that locally express TNF by CNS glial cells, it has been shown that this receptor was able to induce OLG apoptosis, primary inflammatory demyelination and the generation of MS-like plaques [157]. Moreover, EAE mice that only express non-cleavable tmTNF were protected against EAE, suggesting that the interaction between sTNF and TNFR1 mediates the pathology [162]. This concept was further supported by the observation that inhibition of sTNF reduced spinal cord injury [163] and EAE pathology, associated with reduced immunoreactivity, increased expression of neuroprotective and myelin-specific genes and axonal preservation [164,165,166]. Although sTNF inhibition did not prevent OLG loss and demyelination in the cuprizone-induced demyelination model, it induced early remyelination due to improved removal of myelin debris by CNS phagocyting macrophages, indicating that sTNF inhibits the remyelination process [167]. Furthermore, astrocyte-expressed TNF is crucial for blood–brain barrier (BBB) damage and endothelial activation via TNFR1 [114,145,157]. Interestingly, TNFR1 neutralization may indirectly stimulate TNF/TNFR2 signaling in the EAE lesions and promotes remyelination in chemically-induced demyelination [168]. Others successfully applied specific TNFR1 inhibition in EAE using the TNFR1-selective antagonistic mutant TNF protein, PEG-R1antTNF [169] or the commercial monoclonal hamster IgG antibody against mouse TNFR1 [170]. Our research group developed a trivalent TNFR1 Nanobody with very promising characteristics in the EAE pathology [171] (cf. Section 6). MS often presents with memory deficits, and it has been elegantly shown that TNF/TNFR1 signaling in astrocytes is responsible for these cognitive disturbances [172]. However, this symptom has never been therapeutically addressed in mouse studies and the efficacy of TNFR1-inhibiting drugs to overcome memory deficits in MS should definitely be investigated in future research.

TNFR2 is minimally expressed in the CNS in physiological conditions but its expression is boosted in neurological condition in glial cells such as microglia, astrocytes and OLGs [173] but usually not in neurons. In MS, microglia, monocytes and macrophages express TNFR2 and these cell populations also play pivotal roles in the disease [165]. Moreover, TNFR2 null mice subjected to MOG_35–55_-induced EAE showed exacerbated disease, enhanced Th_1_ cytokine production, and enhanced CD4^+^ cell infiltration in the CNS [161]. Conversely, mice only expressing tmTNF (i.e., TNF non-sheddable mice), which mainly signals via TNFR2, were protected during EAE [162]. A neuroprotective role is attributed to TNFR2 because its signaling pathway protects neurons against excitotoxic insults in vitro and in vivo induced by excessive glutamate levels [128,174], and against oxidative stress [168,175]. Furthermore, TNFR2 promotes neuronal survival, OPC maturation and differentiation, OLG proliferation and CNS remyelination [33,168,176,177,178,179]. Given that TNFR2 is also important for T_reg_ expansion and facilitates their suppressive capacity against effector T cells, and since certain T_regs_ are dysfunctional in MS [180], TNFR2′ optimal signaling should be guaranteed. Intriguingly, it has been shown that also TNFR2 on non-hematopoietic cells is important to modulate the fate of T_regs_ and their suppressive functions [181]. However, TNFR2 has a dichotomous function: Gao et al. recently showed that peripheral macrophage/monocyte-TNFR2 drives the immune activation via T cell activation and triggers leukocyte transmigration across the BBB, whereas microglial TNFR2 provides protective signals by promoting anti-inflammatory pathways [173,182]. This should be considered for therapeutic purposes. The complexity is even increased as TNF mediates regression of activated myelin-specific T cells. This prevents prolonged primary myelin reactivity and limits the probability for chronic disease. Although it was first thought that this was mediated by TNFR1, more recent studies pointed towards TNFR2, but also redundancy in this immunosuppressive function is described for the two receptors [88,96,183,184].

See Table 1 and Figure 2 for the involvement of the two TNFRs in neurodegenerative diseases. Collectively, one can say that sTNF/TNFR1 inhibits remyelination, whereas tmTNF directly signals through TNFR2 expressed on OPCs or macrophages to mediate remyelination and to protect against damage.

#### 3.4.3. TNF Involvement in Alzheimer’s Disease

As already outlined in the previous sections, TNF has a very important pluripotent role in the brain. The clinical involvement of TNF in AD is evidenced by the observation that TNF serum and CSF levels are correlated with disease severity and that TNF co-localizes with amyloid beta (Aβ) plaques in the brain. In transgenic mouse models of AD, TNF contributes to disease progression and onset [12]. However, TNF is also a known regulator of synaptic communication between cells. Clearly, the role of TNF in the brain is divergent: low levels of TNF are needed in healthy brains, while overexpression of TNF, primary by microglia, is neurodegenerative. It has been proposed that the synaptic effects of TNF are associated with the synaptic dysfunction that has a central role in AD, particularly with respect to cognitive dysfunction [208]. Indeed, TNF can influence synaptic transmission and plasticity such as hippocampal long-term potentiation (LTP) and synaptic scaling that contributes to early memory loss and learning impairment [192,209,210]. Additionally, it has been demonstrated that TNF contributes to amyloidogenesis, although there is no clear consensus where TNF interferes in the amyloidogenesis process. A study in TNF KO AD mice (5×FAD) found that the diminished amount amyloid plaques and Aβ species are a result of reduced Aβ generation and not a consequence of more clearance [211]. Others state that TNF is implicated in both Aβ generation and clearance [211,212]. TNF, whether or not produced by neurons, promotes the expression of astrocyte beta-secretase 1 (BACE1) and suppresses Aβ clearance by inhibiting microglial phagocytosis [212,213,214]. In contrast, transient hippocampal TNF expression decreases Aβ disposition [215]. In further support of the neurotoxicity of TNF in AD, chronic neuronal TNF overexpression promotes brain inflammation and is detrimental for neuronal viability and these inflammatory events coincides with the appearance of cognitive deficits and synaptic dysfunctions [216]. This suggests that TNF participates in multiple stages of AD pathology [216] (Table 1). Clearly, TNF has again both beneficial and detrimental roles in AD, depending on differences in the (spatiotemporal) TNF expression pattern.

Epidemiological studies demonstrate that the relative AD risk was significantly reduced in RA patients that received the anti-TNF drug etanercept [217,218]. To better understand the contribution of peripheral TNF-mediated inflammation to AD pathology, Paouri et al. crossed AD susceptible mice (5xFAD) with Tg197 mice that have a whole-body expression of hTNF. Interestingly, peripheral hTNF which signals only via mouse TNFR1 [219], robustly activates microglial and astrocytes which in turn clear Aβ but also mediate synaptic loss [185]. Despite of this evidence, preclinical studies in mouse AD models with anti-TNF inhibitors left us with conflicting results. In some studies, the use of TNF inhibitors such as etanercept or infliximab demonstrated clinical benefits [220,221], whereas other failed to reproduce that [222,223]. In addition, results from clinical studies are not always clear: a double-blind randomized trial of peripheral administration of etanercept failed to show cognitive or behavioral benefits [224], whereas perispinal administration of etanercept improved cognitive decline in a small short-term pilot study [225,226]. Intrathecal infliximab administration also improved the cognitive behavior, but this was a case report in one woman thus larger studies are imperative [227]. Recently, a phase I open-label crossover study in patients with mild to moderate AD treated with perispinal administration of etanercept together with dietary supplements could provide more insights into the potential effect of etanercept in AD but unfortunately, results were not conclusive about the cognitive benefit compared to patients treated with nutritional supplements alone [228]. These clinical and preclinical data might indicate that the therapy needs to be initiated at very early stages of AD, rather than in advanced disease, or that a more selective TNF neutralizing approach should be implemented.

In AD brains, TNFR1 protein levels and TNFR1 binding affinity were augmented in contrast to TNFR2 levels and binding affinity compared to non-demented patients [229,230]. Interestingly, our research team found that in the choroid plexus of AD patients, TNF is the main inflammatory upstream mediator, providing detrimental signals via TNFR1. The blood–CSF barrier consists of a monolayer of choroid plexus epithelial cells, and we found that TNFR1 contributes to the morphological damage which is typically seen in the choroid plexus of AD patients [186,231]. Additionally, Li et al. reported that Aβ induces neuronal death via TNFR1 in the AD brain [129] and TNFR1 contributes to the amyloidogenesis via the regulation of BACE1 in APP23 transgenic mice [131]. Our recent study reinforces these results in two AD model: the intracerebroventricular (icv) injection of oligomerized Aβ and in APP/PS1 mice [186]. Our results were in line with observations in APP23 mice devoid of TNFR1 that have less memory deficits, neuronal loss and microglia activation compared to normal APP23 mice [131]. Pharmacological evidence was also provided as inhibition of sTNF, signaling through TNFR1, reduced the accumulation of APP fragments in hippocampus and cortex of 3×Tg-AD mice, and restored synaptic dysfunction and LTP impairment in 5xFAD mice [188,189]. These results were in agreement with the study of Paouri et al. [185]. Strikingly, direct TNFR1 blockage with a TNFR1-inhibiting Nanobody TROS alleviated the AβO-induced memory deficits [186] and the Aβ-mediated inhibition of hippocampal LTP is reversed in absence of TNFR1, providing strong evidence that TNFR1 activation is required for the Aβ-mediated inhibition of LTP [192]. Conversely, inhibition of TNFR2 increased Aβ toxicity in vitro [190] and APP23 mice deficient for TNFR2 displayed exacerbated AD pathology compared to APP23 mice with a functional TNFR2 gene [187]. Furthermore, selective inhibition of neuronal TNFR2 enhanced the Aβ and Tau-related pathologic features in AD and diminished microglia activation needed for Aβ clearance [191]. These observations support the idea that TNFR1 has detrimental roles in AD, whereas TNFR2 needs to be spared to counteract the Aβ-mediated pathology and urges more selective targeting of the TNF pathway (see Table 1). Likewise, in seizure models, TNFR2 is important to protect hippocampal neurons against excitotoxicity and pan-TNF inhibitors that do not spare TNFR2 lead to untoward effects in this brain region, again suggesting that TNFR2 is important in hippocampal repair and neurogenesis [205].

#### 3.4.4. TNF in Parkinson’s Disease

Inflammatory processes are described in PD patients and some may argue that they even trigger the disease onset. This also accounts for peripheral inflammation that enhances the degeneration of dopaminergic neurons. This was clear in animal models, but also human data point in that direction [232]. As in several other neurological disease, increased levels of TNF and sTNF(R1) are evident in the CSF and tissues of PD patients, as well as in postmortem brain tissue. The levels found in serum correlated with disease severity according to some researchers [124,233]. One group also found that *TNF* gene promoter polymorphisms were associated with an earlier age of PD onset [234]. This evidence about the effector role of TNF in PD is strengthened by the observation of very quick fundamental TNF increments in PD mouse models. In addition, TNF was found to be extremely toxic for dopaminergic neurons in vitro. These findings support the idea that the TNF-driven inflammation is essential in the pathogenesis and progression of the disease [12]. Unfortunately, studies in transgenic mice that were subjected to different PD models (injection of parkinsonian neurotoxic agents 6-hydroxydopamine (6-OHDA) or 1-methyl-4-phenyl-1,2,3,6-tetrahydropyridine (MPTP)) have yielded contrasting results. The toxicity of TNF on dopaminergic neurons was demonstrated in TNF KO mice which were less sensitive to MPTP-induced striatal dopaminergic neurotoxicity [235]. As dopaminergic neurons were shown to express TNFR1 and this expression is induced in PD [236], the role of the two receptors was further investigated by several groups. In line with the results obtained in the TNF KO mice, also the double TNFR KO mice were completely protected against MPTP-induced neurotoxicity by suppressing microglial activation [193,194,237]. However, this was rebutted by Leng et al., stating that TNF has protective effects, mediated by TNFR-independent mechanisms [195]. Because the vulnerability of hippocampal neurons to MPTP was increased in mice lacking the two TNFR, a dual and region-dependent role of TNF was proposed and this highlighted the neurotrophic or neuroprotective role of TNF in the hippocampus [194]. This specific role was confirmed by others that observed that TNF does not participate in dopaminergic neuronal cell death in PD but rather alters dopamine metabolism and the survival of dopaminergic terminals [238]. Interestingly, the effects of TNF are not only region-dependent but also dose-dependent: low TNF concentrations in the substantia nigra mediate neuroprotective effects in mice by reducing the nigrostriatal neurodegeneration induced by 6-OHDA although chronic expression of low TNF levels eventually causes dopaminergic cell death, and functionally leads to akinesia. Conversely, high TNF levels induce progressive neuronal cell loss accompanied by gliosis and inflammation [239,240]. In addition, in preclinical studies, contradictory results were obtained as early TNF blockage worsened the outcome after intrastriatal 6-OHDA injection [241] but intranigral infusion of sTNF neutralizing therapeutics attenuated the nigral dopaminergic loss and microglia activation [196,197,198]. This sTNF inhibitor could cross the BBB and had disease-modifying properties upon peripheral administration [198]. Inhibition of TNF synthesis by thalidomide partly protected against MPTP-induced dopamine depletion [235]. Interestingly, a BBB-penetrating Trojan horse has been designed consisting of a TNF decoy receptor fused to a mAb against the mouse transferrin receptor (TfR). This drug was neuroprotective in the 6-OHDA mouse model of PD. In contrast, etanercept that does not penetrate the BBB had no effect on the neurobehavior [242]. To analyze and interpret these incoherent results, one should account for the divergence across all studies, with differences in model, doses and timing of analysis [243]. Furthermore, these models are difficult to extrapolate to human situations as the TNF peak in these mouse models is relatively short, whereas the levels remain elevated along the disease course in PD patients.

## 4. TNF Inhibitors

### 4.1. Approved TNF Inhibitors

The initial concept to use recombinant TNF as an anti-tumor agent was quickly followed by the idea to consider TNF as a drug target for inflammatory diseases [244]. Indeed, TNF represents an active and attractive objective for drug development despite the initial skepticism because of the failure of anti-TNF drugs in sepsis patients [245,246]. The rationale to target TNF was first confirmed in a murine RA model [247], and, in 1993, RA patients were successfully treated with mAb cA2, later known as infliximab [248]. This success was the start to further develop anti-TNF drugs in TNF-involving inflammatory diseases. Currently, five anti-TNF biologics and in total 25 drugs that inhibit or modulate the effects of TNF, are approved for clinical use by the Food and Drug Administration (FDA) and European Medicines Agency (EMA) for the treatment of RA, AS, psoriasis and psoriatic arthritis (PsA), juvenile idiopathic arthritis (JIA), CD and ulcerative colitis (UC). Recently, adalimumab was also licensed in some countries to treat uveitis and hidradenitis suppurativa which is a chronic skin disease characterized by recurrent abscesses. Furthermore, there is off-label use in Behcet’s syndrome and amyloidosis [245,249]. The introduction of TNF inhibitors on the market has revolutionized the treatment of these pathologies and anti-TNF therapy is now standard of care for RA. Moreover, these blockbusters currently belong to the top-10 best-selling drugs in the world, with adalimumab being the world’s best-selling medicine, counting for US $10 billion per year and the total sale of the various anti-TNF drugs exceeds US $25 billion [245]. Currently, another 151 TNF inhibitors are in the clinical pipeline.

**Figure 3:**
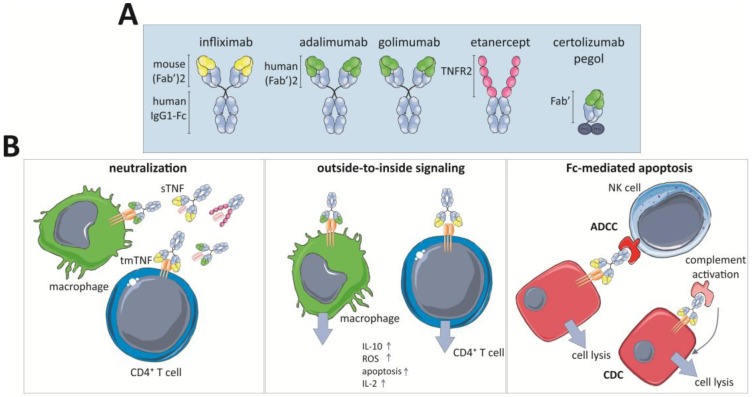
Structure (**A**); and mechanisms of action (**B**) of anti-TNF biologics. All anti-TNF biologics neutralize membrane-bound (tmTNF) and soluble TNF (sTNF) but, in addition to that, some inhibitors also induce outside-to-inside signaling via tmTNF and their Fc-regions mediate antibody-dependent cellular cytotoxicity (ADCC) and complement-dependent cytotoxicity (CDC). ADCC: antibody-dependent cellular cytotoxicity; CDC: complement-dependent cytotoxicity, Fab: fragment antigen binding; IgG: immunoglobulin G; IL: interleukin; NK: natural killer; ROS: reactive oxygen species tmTNF: transmembrane TNF.

Three of the five approved TNF-inhibitors are full-length monoclonal antibodies (mAbs): infliximab (Remicade^®^ and biosimilars Remsima^®^, Inflectra^®^, Flixabi^®^, and Ixifi^®^), adalimumab (Humira^®^ and Cyltezo^®^, Imraldi^®^, Amgevita^®^, and Solymbic^®^) and golimumab (Simponi^®^). Next to these, certolizumab (Cimzia^®^) and etanercept (Enbrel^®^ and biosimilars Erelzi^®^ and Benepali^®^) are also approved. Although they all neutralize the TNF activity, they each have different characteristics and routes of administration. Furthermore, all of them are equally effective against RA, but not against CD. These discrepancies are attributable to different mechanisms of actions that are not completely understood [38,250,251] (Table 2 and Figure 3).

In 1998, infliximab was the first TNF-targeting antibody approved in the US to treat CD and later UC. It is a chimeric monoclonal IgG1 Ab that comprises a human constant domain and murine variable regions. The infliximab biosimilar CT-P13 (Remisma^®^ or Inflectra^®^) is highly similar to its originator and therefore clinically used in the same way [252]. Golimumab and adalimumab are full human Abs that were produced by recombinant DNA technology and certolizumab is a humanized Fab’ fragment that is conjugated to polyethylene glycol (PEG) to increase the serum half-life. This reduces the requirement for frequent dosing and possibly reduces the immunogenic nature. Finally, etanercept is a fusion protein of the extracellular domain of human TNFR2 receptor coupled to the Fc region of human IgG1. Etanercept binds circulating sTNF and acts as a decoy receptor that prevents TNF-interaction with the cell surface receptors.

### 4.2. Mechanisms of Action of TNF Inhibitors

All anti-TNF agents have the same target but not all of them are equally efficacious in all considered diseases, suggesting that different working mechanisms are inherit to certain antibody structures. It is clear that particularly in CD alternative effector mechanisms rather than pure TNF neutralization account for their efficacy whereas this is less the case in RA in which all marketed anti-TNF drugs are indicated. First, their affinities for TNF are different and generally tmTNF is neutralized with lower affinity than sTNF [252]. Etanercept is the only one that is capable of neutralizing lymphotoxin-α (LT-α) and it only neutralizes trimeric TNF. However, TNF inhibitors show equal sTNF neutralizing potency [276]. Neutralization of sTNF or tmTNF blocks the TNF-mediated activation of TNFRs and this results in suppression of inflammatory mediators. Indeed, TNF recruits pro-inflammatory immune cells (cf. Section 3.2) and this process is thus abrogated by the drugs. Use of the TNF inhibitor also reduces intestinal permeability in CD through decreased intestinal epithelial cell apoptosis [276].

The TNF blockers’ ability to crosslink tmTNF can be different, e.g., infliximab forms more stable complexes with tmTNF than etanercept. Consequently, binding of infliximab to tmTNF can activate the “outside-to-inside signaling” or reverse signaling, and, in that case, TNF is considered as a receptor rather than a ligand (Figure 3). As a direct consequence of this interaction, apoptosis is induced in the tmTNF-expressing immune cells and this was proposed as one of the mechanisms of action in CD. This mechanism also impairs their production of pro-inflammatory mediators. Interestingly, apoptosis can also indirectly be induced in immune cells upon anti-TNF treatment. In CD, there is an anti-apoptotic signal induced by the interaction between monocytic tmTNF and TNFR2 expressed by CD4^+^ T cells. This mechanism is critical for granulomatous inflammation seen in CD, but this interaction is inhibited by anti-TNF resulting in lamina propria T cell apoptosis [277,278]. As the affinity for tmTNF is not similar among the different anti-TNF drugs, clinical features against this type of inflammation are not equal as well. Indeed, etanercept cannot activate reverse signaling via tmTNF which might explain its inefficiency in CD [13,279]. Thus, these drugs might stimulate apoptosis by reverse signaling or impairing apoptosis by abrogation of the TNF/TNFR signaling pathway. Importantly, TNF can also mediate other forms of cell death such as necroptosis [22,122] (cf. Section 2). Consequently, blockage of TNF might also impair this inflammatory process which might also account for the efficiency of the drugs. However, to our knowledge, not much research has been done about this topic.

In addition to their direct TNF-related capacities, the TNF-inhibitors have a panoply of other effects although currently not all their molecular mechanisms of action are completely understood [252,276]. Infliximab, adalimumab and golimumab are the only full-length mAbs and thus they also possess Fc-effector activity in addition to their general TNF-blockage properties. As a result, they can induce antibody-dependent cellular cytotoxicity (ADCC) and activate the complement pathway leading to cell-dependent cytotoxicity (CDC) and apoptosis (Figure 3). Etanercept contains a truncated Fc-domain without the CH1 domain of IgG1, therefore it induces ADCC and CDC but to a lower extent than the mAbs [252]. Certolizumab pegol, being a Fab’ fragment, is due to its structure incapable of inducing ADCC and CDC and therefore its working mechanism does not rely on the complement pathway [280] (Figure 3).

In IBD, also the interplay between the IgG1-Fc domain of the anti-TNF antibodies and the Fcγ-receptors (FcγR) on macrophages accounts for the efficacy of the anti-TNF antibodies by increasing the number of regulatory CD206^+^ macrophages upon activation. This M2-type macrophage subset expresses specific membrane markers and inhibits T cell proliferation [281]. Alternatively, adalimumab promotes the interaction between monocytes and T_regs_ via TNFR2 in RA. Adalimumab enhances the expression of tmTNF in monocytes upon binding which improves the interaction between tmTNF and TNFR2 on T_regs_ and boosts their suppressive activities [282]. In addition, infliximab gives rise to a CD4^+^CD25^hi^FoxP3^+^ T_reg_ population that restrains pro-inflammatory cytokine production. This newly generated T_reg_ population compensates for the natural T_reg_ pool that is defective in autoimmune diseases such as RA [283].

The anti-TNF inhibitors were investigated in many observational studies as well as in open-label extensions of the original double-blind trials and in post-marketing observational studies. These studies provided data about the long-term efficacy and safety of the drugs. Generally, the anti-TNF inhibitors were found to be well-tolerated and to improve health-related quality-of-life (QoL) outcomes in the aforementioned diseases [284]. For RA, anti-TNF drugs are now standard-of-care, initiated after failure of treatment with the immunomodulator methotrexate (MTX) in patients. In most of the RA cases (70–80%) TNF-inhibitors are used as combination therapy with MTX. Systematic reviews of clinical trials demonstrated an additional effect of this combination in RA [245,285], whereas for CD the results from clinical trials comparing monotherapy with combination therapy were conflicting [286]. It should be noted that the risk for adverse outcomes is possibly increased with combination therapy. The combination of etanercept with MTX has similar efficacy in the therapy of RA as infliximab and adalimumab, while it is not active against CD.

### 4.3. Pitfalls of TNF Inhibitors

**Figure 4:**
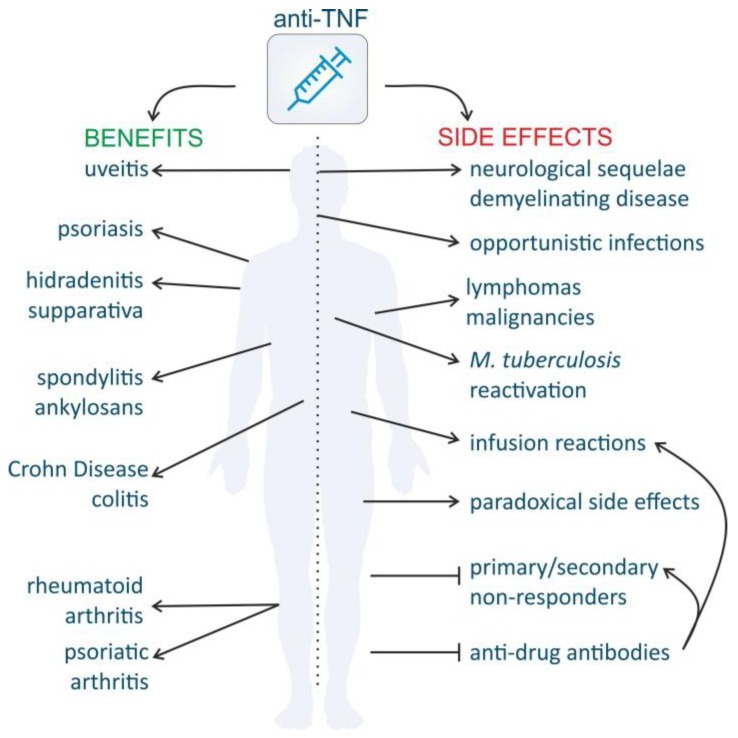
Beneficial and side effects of anti-TNF medication. In addition to the well-known beneficial effects in several autoimmune diseases, anti-TNF medication is associated with many side effects.

The introduction of TNF-antagonists for treatment of inflammatory disorders substantially improved the QoL of the patients, and in IBD it also reduced the number of surgeries and hospitalizations. However, the long-lasting use of these drugs coincides with a number of important adverse events (Figure 4) [287].

#### 4.3.1. High Costs

Anti-TNF drugs are real blockbusters for the pharmaceutical companies because this is the best-selling pharmaceutical drug class with sales over US $25 billion. This puts a monumental pressure on health care systems, meaning that many countries even cannot afford a decent policy around these drugs. A retrospective study performed in 2016 estimated the annual cost of the use of biologics per patients in the USA. The most used biological was etanercept (48%), followed by adalimumab (29%) and infliximab (12%) and the annual costs per treated patient were US $24,859 for etanercept, US $26,537 for adalimumab and US $26,468 infliximab [288].

#### 4.3.2. Clinical Response

Although many patients benefit from treatment with anti-TNF drugs, a big problem in the clinic remains the high number of patients that do not respond to the therapy: 13–40% of patients fail to respond to initial anti-TNF therapy (primary non-responders) and up to 50% of patients lose responsiveness during therapy (secondary non-responders) [289]. Primary non-response is defined by the lack of improvement of clinical signs and symptoms with induction therapy. Loss of clinical remission frequently occurs in CD patients treated with anti-TNF drugs and when the treatment fails the therapeutic options are often limited [290]. Therefore, early identification of patients at risk is of major clinical importance. Whether a patient responds well to the initiated therapy depends on multiple clinical (e.g., disease phenotype and response to previous therapies), genetic and immuno-pharmacological variables [291]. In IBD patients, mucosal healing is not obtained in 50% of the patients treated with anti-TNF biologics, and therapeutic efficacy is shown to be dependent on the interaction between the Fc region of the anti-TNF IgG and the cellular FcγR. Recently, a hypo-fucosylated form of adalimumab was designed and was found to have improved mucosal healing properties thanks to its higher affinity to FcγRIII and induction of CD206^+^ macrophages [281]. Consequently, some studies correlated the low-affinity FcγRIIIa allotype in IBD patients with lower changes to respond to therapies with IgG1 Ab infliximab and reduced mucosal healing [292]. Currently, elaborate studies are ongoing to identify inflammatory biomarkers that allow the stratification of patients into responders and non-responders. Some biomarkers seem promising, although they still have to be validated in large patient cohorts to verify their specificity and reliability to predict the response in the clinic [252,291,293].

Secondary non-responsiveness can be explained by the formation of anti-drug antibodies (ADAs) (e.g., anti-anti-TNF antibodies) in a subset of patients and the risk of loss-of-response is increased by at least threefold when ADAs are present [291] (Figure 4). ADAs were not only found against the chimeric mAb infliximab, but also against the fully humanized mAbs adalimumab and golimumab. This is possibly a consequence of the interaction between anti-TNF and tmTNF on antigen presenting cells and the subsequent rapid internalization. The internalized anti-TNF will be processed and its peptides displayed on the surface of the APCs will mount a T cell proliferation response [294]. Unfortunately, technical factors, standardization of the assays used to determine ADAs, and the timing of the measurements makes this a complex subject to investigate. The neutralizing ADAs that inhibit the functionality of the biologics are only a subset of the ADAs that can be found in patients. Neutralizing ADAs are generally directed against the biological active site of the drug, e.g., antigen binding part of the drug. The loss-of-response elicited by neutralizing ADAs can be countered by dose-escalation or by switching to another anti-TNF drug, but sometimes the therapy needs to be discontinued [295]. A good drug compliance and concomitant treatment with immunosuppressive agents demonstrated a reduction in the occurrence of ADAs in multiple clinical trials and improved clinical outcomes [276,296]. In addition, co-treatment with MTX was found to be beneficial in that regard [286]. ADAs may form immune complexes that are rapidly cleared by the reticulo-endothelial system and are associated with decreased drug levels, short duration of response and higher risk of infusion reactions and even acute hypersensitivity (anaphylaxis) [297,298]. Luckily, the impact of the ADA is inversely correlated with their frequency of occurrence, meaning that binding ADAs are generally more common than neutralizing ADAs or ADAs that form immune complexes. Surprisingly, the presence of ADAs may be permanent but may also be transient, appearing in one single measurement without recurrence [299].

To reduce the risk of loss-of-response, therapeutic drug monitoring (TDM) has become standard of care in the clinical setting for many clinicians, because there is a well-established correlation between serum trough levels of the drugs and clinical response [291]. Indeed, adaption of the dose based on the trough levels were more effective at inducing remission in IBD patients than clinic-based dosing [300]. TDM allows increasing the dose in patients with sub-optimal drug levels and this leads to better clinical effects. Moreover, it is widely believed that sub-therapeutic doses contribute to the development of ADAs, further highlighting the importance of TDM. Dose de-escalation is done in patients with supra-optimal levels, leading to lower drug exposure and reduced costs without impact on the clinical response [301,302].

#### 4.3.3. Increased Susceptibility to Infection and Malignancies

The beneficial effects of anti-TNF medication are undeniable but there are serious concerns considering their safety. In addition to acute problems, such as infusion reactions, other severe adverse events might occur (Figure 4). Because TNF has an important function in host defense and in the protection against (intracellular) bacteria, as discussed in Section 3.2, infectious complications due to shutdown of this arm of the immune system are a big concern in patients treated with anti-TNF drugs. Post-marketing data have revealed a rate of 0.69% serious infections and also a drastic increase of activated tuberculosis with aberrant granuloma formation was reported [303,304]. These unwanted effects are considered as a class effect, because all anti-TNF drugs appear to have an equally high risk in acquiring new tuberculosis infections, although the mAbs seem to cause more infections with reactive latent tuberculosis [304]. This serious health issue initiated the recommendation to screen for tuberculosis with the QuantiFERON Gold Test and to treat infections, even when they are latent, before initiating the anti-TNF treatment [305]. Since then, the number of reports on tuberculosis infections has decreased, but other untypical opportunistic viral and fungal infections have popped up, including cytomegolavirus infection, *Pneumocystis jirovecii* pneumonia, histoplasmosis and aspergillosis. Risk factors to develop these infections are age and concomitant treatment with corticosteroids, and the overall risk of these infections should be considered before the treatment is started [306].

Regarding the risk for malignancies, studies in mice attributed an important role to TNF in the process of tumor immune surveillance [307]. However, TNF KO mice do not spontaneously develop tumors, not even in a susceptible background [38]. Notwithstanding this observation, serious concerns about anti-TNF drugs in human patients remained. Earlier studies reported an increased risk for lymphomas (Hodgkin’s lymphoma, B cell lymphoma, etc.) and other malignancies, but more recent studies and registry databases found no association between anti-TNF treatment and the occurrence of solid or hematologic cancers [308]. However, it is possible that significances are unclear because the studies are underpowered due to the low incidence of these adverse effects. In addition, because the relatively short period of clinical use of the anti-TNF drugs and the long time it may take for tumors to develop, it may be too early to make good and relevant association and risk studies. Anyway, in the case cancer is found during anti-TNF treatment, it is advisable to interrupt the treatment until the cancer is under control [309]. Noteworthy to conclude, patients that use anti-TNF medication for chronic inflammatory disease are already at higher risk for infections and malignancies for several disease-related reasons, regardless of their treatment [308,310].

#### 4.3.4. Demyelinating Disease and Other Neurological Side Effects

Neurological side effects have been reported and recent data suggest a role for anti-TNF drugs in the induction of neurological disease, especially demyelination of the CNS as well as implications at the level of the peripheral nervous system (Figure 4). The prevalence of these side effects has been estimated to range between 0.05% and 0.2% for infliximab, etanercept and adalimumab [311]. Eighty percent of the reports about CNS demyelination are about optic neuritis, but also cases of MS or MS-like diseases have been reported. In addition, peripheral nervous system disorders were documented, such as Miller Fisher syndrome, Guillain-Barré syndrome and other neuropathies [312]. There are several hypotheses to explain the possible relationship between TNF-antagonists and demyelination but none of them is believed to be adequate [313]: (1) The occurrence of demyelinating disease could be attributed to the unmasking of a latent pre-existing form of MS, to the emergence of a new demyelination episode or to incidental coexistence of the two disorders. (2) The administration of anti-TNF agents could unmask a latent infection that is critical for the development of MS [311]. (3) Local TNF production in the CNS by pathogenic T cells induces demyelination and, therefore, the demyelination seen with TNF-antagonists may look paradoxical. However, the presence of CNS barriers, e.g., the blood–CSF and BBB, renders CNS access almost impossible as the biologics have a size of approximately 150 kDa. Even though the permeability of the barriers is increased in inflammatory conditions, this does not lead to significant blood-derived protein increment, and by inference anti-TNF biologics, in the CSF [313]. In patients, infliximab was not detected in the CSF, even in presence of active MS and BBB impairment [152]. (4) Prolonged blockage of peripheral TNF increases the T cell response to a specific antigen. This may lead to a significantly increased amount of highly activated myelin-specific autoreactive T cells, ultimately exacerbating autoimmune demyelinating diseases [313]. (5) Anti-TNF drugs can neutralize TNF systemically but not within the CNS. This results in an overall reduction in TNF in the body but relatively unchanged TNF levels in the brain. This creates an artificially high local concentration of brain (the “sponge” effect) leading to local tissue injury. Either way, once unexplained neurologic symptoms appear, the anti-TNF treatment should be discontinued [312,313].

In addition to these neurological disorders, CD patients treated with anti-TNF drugs reported fatigue which was significantly associated with the use of these drugs. Subgroup analyses also indicated that long-term therapy duration and combination without azathioprine were risk factors for the occurrence of fatigue [314,315].

#### 4.3.5. Paradoxical Side Effects

In addition to the “common” adverse effects, there are also paradoxical side effects described in patients treated with anti-TNF drugs (Figure 4). They represent the unexpected onset or exacerbation of an autoimmune disease for which TNF blockers are indicated other than the one the patient is treated for. These disorders are mainly reported in patients with rheumatic diseases and IBD [316]. Psoriasiform skin reactions are the most frequently observed dermatological adverse effects seen in patients treated with anti-TNF therapy, but also uveitis, vasculitis, Graves’ disease and granulomatous diseases such as sarcoidosis have been reported [316,317,318,319,320]. Psoriatic skin reactions mostly occur about five months after first exposure to TNF blockers. Large cohorts also reported that RA patients treated with etanercept for juvenile idiopathic arthritis, AS or RE developed new-onset CD. Another common paradoxical side effect is the appearance of autoantibodies and a subset of patients developed drug-induced lupus erythematosus (DILE) [321]. SLE is a heterogeneous disease characterized by the production of autoantibodies that form immune complexes leading to inflammation in various organs. TNF is involved in the pathology of SLE but an open-label study showed that TNF inhibition by itself led to the paradoxical formation of autoantibodies [322]. Unfortunately, these adverse effects are probably underreported and the mechanisms unclear. There are indications that an imbalance of cytokines towards IFNs, chemokines and probably IL-17 is implicated in the pathogenesis [323,324]. The first hypothesis attributes a central role to type I IFN-α, which is highly implicated in psoriasis [320]. TNF downregulates the production of IFNs by the plasmacytoid DCs, thus TNF inhibition would enhance IFN production thereby favoring psoriasis development [320]. In addition, an imbalance in cytokines of the IL-12/IL-23 pathway via activation of the Th_17_ pathway is proposed as a possible mechanism [325]. A third hypothesis accounts for the impairment of the TNF-induced apoptosis of autoreactive T cells by the anti-TNF drugs. This lack of autoreactive T cell destruction induces new or aggravated forms of autoimmunity. In these cases, boosting or restoring TNF activity might be therapeutic [326]. These paradoxical side effects appeared between one month and one year after initiation of the therapy. When these symptoms appear in patients, withdrawal of the treatment reverses this unwanted effect in nearly 75% of the cases [316]. As this side effect is considered as a class effect that is seen with all TNF inhibitors, switching to another inhibitor is mostly not helpful.

## 5. Other Anti-TNF and TNF-Modulating Drugs

### 5.1. TNF Inhibitors

In addition to the well-known approved anti-TNF inhibitors, there are several other anti-TNF drugs developed or under development (Table 2). In China, a phase I clinical trial is completed with a humanized anti-TNF mAb *SSS-07* against RA, but no results are provided yet (NCT02460393). The bovine polyclonal milk-derived anti-TNF Ab *AVX-470* can be administered orally thanks to the stability of bovine Igs in human intestinal secretions. After oral administration, this Ab remained localized in the gut, and in a double-blind, placebo controlled study AVX-470 appeared to be safe and well tolerated, and was associated with dose-dependent increases in clinical and endoscopic remission in patients with active UC (NCT01759056) [262,263]. The involved company Avaxia Biologics has created orally administered anti-TNF mAbs, Avaximabs^®^, that are stable in the gastro-intestinal tract. These will be explored for the treatment of necrotizing enterocolitis (NEC). Unfortunately, clinical drug development goes along with many failures too: *CDP571* (Humicade^®^), a humanized mAb against TNF failed to demonstrate clinical efficacy for sparing steroids in CD patients and further development was discontinued [264]. Other approaches than Abs are also considered, as illustrated with the clinical success of the Nanobodies (Nbs) [327]. A highly promising Nb is generated by Ablynx against TNF and is called *ozoralizumab* [328]. This drug is now under clinical investigation to treat autoimmune diseases and proof-of-concept was already obtained in a phase II RA study [265]. Another bivalent Nb was also engineered consisting of two monomeric variable domains of heavy-chain only Abs (VHHs). The construct *VHH#1-3* antagonizes the binding of TNF to its receptors with picomolar potencies. As this drug has a different mode of binding, i.e., it can bind a single trimeric TNF and blocks two of the three receptor binding sites of TNF, it distinguishes itself from other TNF neutralizing drugs [266]. The ease of cloning and production allows Nbs to be locally secreted by the genetically modified probiotic *Lactococcus lactis* after oral administration, as was done with an anti-TNF Nb [329]. Preclinically, this innovative approach was efficacious in colitis without causing immunogenicity and is under clinical investigation. In addition, variable new antigen receptor (VNAR) domains against TNF were developed, originated from immunized sharks. Multivalent VNARs neutralize TNF at picomolar concentrations and were as efficacious as adalimumab in in vitro models of intestinal epithelial barrier dysfunction. Therefore, these drugs could be considered as a novel alternative class of biological agents [330]. Similar to etanercept, *Lenercept* is a soluble fusion protein consisting of TNFR1 fused to the hinge region of the IgG1 Fc region. It entered clinical trials for indications such as sepsis, RA and MS. However, the clinical trial for treatment of RRMS had to be terminated due to unforeseen exacerbations of the symptoms [53,153]. *Hitanercept* is a variant of etanercept that carries a mutation in the TNFR2 domain of the fusion protein and exhibits higher affinity to sTNF and tmTNF than etanercept [269,331]. In the CIA model for RA, hitanercept is more efficacious compared to etanercept. Interestingly, hitanercept is also more potent to induce reverse signaling via tmTNF and to mediate CDC and ADCC. Therefore, the drug also has therapeutic potential in CD and UC. Currently, the tolerance, pharmacokinetics and preliminary efficacy of hitanercept in RA are assessed in a phase I clinical trial in China (NCT02481180) [269]. *HL036* is a small TNFR1 fragment (19 kDa) with enhanced ocular tissue penetration. This drug is formulated as an ophthalmic solution and is currently under clinical investigation (phase II) for dry eyes disease (NCT03334539) after it was shown to be safe in healthy volunteers. In addition, other formulations for inflammatory ocular diseases are currently considered. *Onercept* is a PEGylated form of soluble human TNFR1 that was tested in psoriasis and CD. Despite promising early clinical results, onercept proved not to have an exceptional efficacy and safety profile in both diseases and therefore further development was stopped [267,268,332,333]. Another potentially interesting candidate that is based on the same rationale as onercept is *pegsunercept*, a PEGylated soluble TNFR1. It has been tested for RA, but also here, development was discontinued and this decision was based on recommendations of two separate independent Data and Safety Monitoring Boards [271]. Likewise, the development of the polyclonal anti-TNF Fab fragment *Azd9773* (CytoFab^®^) was suspended as the drug failed to show efficacy in severe sepsis and septic shock [270]. Another new therapeutic approach relies on the active immunization with *TNF-kinoid* or with *CYT007-TNFQb*, inducing endogenous polyclonal anti-TNF antibodies that neutralize circulating TNF in inflammatory immune-mediated diseases. With TNF-kinoid, proof-of-concept was obtained in a mouse RA model and these findings were translated into the clinic in patients that experience secondary non-responsiveness of TNF-antagonists. A phase Ia clinical trial showed that therapeutic vaccination induced dose- and schedule-dependent anti-TNF Abs in RA patients and was well tolerated. Moreover, patients with anti-TNF Abs showed a trend towards clinical improvement [272]. The drug was also investigated to treat CD patients, and a high clinical response was reported with remission rates in half of the patients. However, the clinical efficacy needs to be weighed against the potential harmful consequences of life-long ablation of TNF and probably for that reason further development was suspended [273]. The same holds true for *CYT007-TNFQb* of which the phase I/II clinical trial was discontinued in psoriasis patients [274]. Progranulin is an endogenous glycoprotein expressed in neurons and glia cells that directly interacts with TNFR1 and TNFR2. Proganulin has anti-inflammatory activities by the inhibition of the TNF activity, and *Atsttrin* is a progranulin-derived engineered protein that showed efficacy against RA and osteoarthritis in preclinical models [334,335]. Up until now, no clinical trials are reported, but also this candidate may be an interesting alternative to the generally used anti-TNF biologics. The small chemical triazoloquinoxaline inhibitor *R-7050* is a TNFR complex inhibitor that improves the outcome upon intracerebral hemorrhage, suggesting its use as adjunct therapy in the treatment of neurological injury [336]. The drug does not interfere with TNF-TNFR1 binding, but acts via the inhibition of receptor-adaptor molecules complex formation and subsequent receptor internalization [336]. In addition, interesting is the approach exploited by the group of Pardridge. They engineered a BBB-penetrating TNF inhibitor by fusion of the extracellular domain of TNFR2 to a chimeric monoclonal antibody against the mouse TfR. This Trojan horse approach led to rapid therapeutically relevant amounts of drug in the brain following intravenous, subcutaneous and ip administration, and was protective in mouse models of PD, AD and ischemic stroke [223,242,275,337]. Finally, a novel chemically synthesized anti-TNF compound is described. *C87* is an TNF-TNFR interaction modulator as it directly binds to TNF and prevents TNFR signaling and subsequent Casp-8 and NF-κB activation. It was found from an initial screen of ~90,000 compounds and has in vivo potency. The only remaining challenge is to determine toxicity and stability with longer-term use [338].

### 5.2. TNF Modulators

Other less specific anti-TNF agents are thalidomide and its derivatives lenalidomide and pomalidomide, curcumin and minocycline. Initially, thalidomide was indicated as an effective tranquilizer and painkiller associated with enormous teratogenic side effects in human. Thalidomide is now recently re-introduced as well-known (non-specific) TNF-inhibitor as it reduces the rate of TNF synthesis by enhancing the degradation of the transcript. Currently, thalidomide is under investigation to treat neurodegenerative disorders that implicate TNF-signaling such as AD, PD and amyotrophic lateral sclerosis (ALS) as this small-drug molecule can penetrate into the brain [235,339,340,341,342]. The broad-spectrum tetracycline antibiotic drug *minocycline* decreases TNF synthesis in addition to its bacteriostatic and anti-inflammatory actions. Additionally, it also inhibits MMPs, reduces cyclooxygenase 2 (COX-2) activity and prostaglandin E2 production, and attenuates apoptosis [12]. In PD models, minocycline attenuated MPTP-induced microglia activation, but could not abolish the neurotoxicity [237]. By contrast, *curcumin* (diferuloymethane) is a natural anti-inflammatory agent that inhibits TNF transcription at several levels, but mostly via inhibition of NF-κB. Consequently, curcumin also antagonizes other pro-inflammatory cytokines including IL-1β and IL-6. It is a broad-acting anti-TNF that can be orally consumed via natural food spices. Unfortunately, it is poorly soluble in water and has a poor bio-availability [343]. Currently, neuroprotective characteristics are attributed to curcumin and therefore it is under active investigation for AD amongst others [344]. In addition, xanthine derivate pentoxifylline and bupropion have shown to decrease TNF synthesis [345]. By increasing the signaling at beta-adrenoreceptors and D1 receptors, bupropion increases cyclic AMP (cAMP) which subsequently inhibits TNF synthesis [346]. Unexpectedly, a novel crosstalk pathway between neural and immune receptors was found as several 5-hydroxytryptamine (HT) agonist hallucinogens (such as (R)-DOI, TCB-2, LSD and LA-SS-Az) are potent TNF inhibitors, with DOI being the most potent one. This indicates that activation of the serotonin 5-HT(2A) receptors represents a novel potential therapeutic avenue for TNF-involving disorders [347,348].

## 6. A New Chapter of Inventive TNF Manipulating Approaches

The anti-TNF drugs on the market were based on the wide perception that TNF is a pathological factor, ignoring the fact that TNF can also have beneficial and unique indispensable properties e.g., in immune regulation and tissue regeneration, as discussed above [33,93,102,114,349,350]. This is also illustrated by the numerous side-effects that are inherent to long-lasting TNF blockage. Therefore, more discriminative approaches hold the potential to increase the safety and efficacy of the drugs. Indeed, as outlined in previous sections, especially for neurological diseases more selective approaches are warranted as there is a clear discrepancy between TNFR1 and TNFR2 signaling in the brain typified by the aggravated disease symptoms that are induced by anti-TNF drugs in MS patients. Nonetheless, also non-neurological diseases will benefit from receptor-discriminatory drugs as pan-TNF neutralization induces neurological phenomena in some patients.

### 6.1. Selective TNFR1 Targeting

Given that TNFR1 and TNFR2 mediate different cellular effects, it is interesting to selectively target one of the two. This field is currently actively explored via several approaches [97]. ATROSAB is a human TNFR1-specific antibody that demonstrated to efficiently block the activity of TNF and LT-α in vitro [351,352]. Williams et al. described a monoclonal hamster IgG against mouse TNFR1 that was effective to reduce symptoms in EAE [170], and also a TNFR1 selective antagonistic mutant TNF protein, PEG-R1ant-TNF has been described to have this property [169]. PEG-R1ant-TNF was also effective in the CIA model for RA and against arterial inflammation [353,354]. The efficacy of a single TNFR1-binding domain bispecific antibody, MDS5541, was evaluated against RA in vitro in synovial membrane cell cultures from RA patients and in vivo in the CIA model of RA [107,355]. Selective TNFR1 inhibition established with this drug led to the expansion and activation of T_regs_ with upregulation of FoxP3-dependent genes. This effect again highlights the importance of preserving the TNF/TNFR2-mediated signaling pathway. In addition, two fully human anti-TNFR1 single domain antibodies have been developed and were investigated by GlaxoSmithKline [356]. The first, GSK1995057 attenuated lung injury in different preclinical models of acute respiratory distress syndrome [356,357]. However, in a phase I clinical trial with this small inhibitor, infusion reactions arose in healthy volunteers because of the presence of naturally occurring pre-existing ADAs [358,359]. Consequently, a new trial was initiated in which only healthy subjects prospectively demonstrated to be seronegative for the pre-existing ADAs were eligible for participation (TFR116343). In these subjects, nebulized GSK1995057 prevented acute lung injury in an LPS-induced model. The drug will now be evaluated in a phase IIa clinical trial [360]. To deal with the problem of pre-existing ADAs, a second single domain was designed (GSK2862277). In a phase I trial, this drug was well tolerated by both the inhaled and iv route [359]. A placebo-controlled randomized phase II trial was set up in patients that undergo oesophagectomy surgery and that were at risk to develop acute respiratory distress syndrome. The drug GSK2862277 was administered as an orally inhaled aerosol pre-operative, but the trial was terminated earlier as the study met the designed stopping criteria (NCT02221037). However, GSK still concludes that selective antagonisms of TNFR1 using inhaled drugs might offer therapeutic benefit in patients with acute respiratory distress syndrome. Another interesting approach to selectively target TNFR1 is by interfering with the PLAD association, necessary for TNF/TNFR1 signaling [16]. Targeting the TNFR1 PLAD domain has already been proposed by several groups as a promising strategy in autoimmune diseases such as diabetes and RA [361,362]. The marketed anti-asthma drug zafirlukast also disrupts the interaction between the TNFR1 PLAD domain and is thus considered as a selective TNFR inhibitor [363].

Importantly, our research group also generated a trivalent human TNFR1 inhibiting Nanobody consisting of two paratopic TNFR1 binding Nbs linked to an anti-albumin Nb. This Nb only binds to human TNFR1 without being cross reactive for the mouse homologue. One of these Nbs also competitively inhibited the TNF/TNFR1 signaling, but the potency of this Nb was improved after incorporation in the TROS construct. This construct effectively inhibited TNFR1 signaling in vitro and ex vivo on isolated colon biopsies from CD patients [328]. Proof-of-concept of this promising molecule has been first delivered in humanized mice carrying a human *TNFRSF1A* gene, subjected to the mouse EAE model of MS. In these transgenic mice Prophylactic as well as therapeutic ip administration of the drug prevented or halted disease development, respectively [171]. The drug prevented demyelination and treatment with TROS maintained the expression of several important neuroprotective genes that are downregulated in MS patients. Because choroid plexus TNFR1 is also an important detrimental mediator in AD as was recently shown by the group, also the possibility to inhibit TNFR1 therapeutically with TROS was investigated in the acute AD model of icv oligomerized amyloid beta (AβO) injection. Strikingly, TROS prevented the AβO-induced memory decline in these mice after icv injection, confirming the therapeutic possibilities of this molecule [186].

### 6.2. Selective TNFR2 Targeting

The opposite approach that is currently investigated implies TNFR2 activation to stimulate the TNFR2-mediated protective pathways in autoimmunity and neurodegenerative diseases [364]. It has been suggested that this strategy might be superior to TNFR1 antagonism because of the restricted cellular expression of TNFR2. TNFR2 is not only considered as a costimulatory receptor for T cells and critically involved in the development of T_regs_, recent studies also provide new insights into the role of tmTNF/TNFR2 signaling in the suppressive activity of myeloid-derived suppressor cells (MDSCs). Indeed, MDSCs require membrane TNFR2 expression to exert optimal suppressive activity [365,366]. Interestingly, chronic inflammation increases the sensitivity of these suppressive cells for TNFR2 costimulation [367]. Efficient TNFR2 activation requires oligomerization of TNFR2 by tmTNF. Alternatively, oligomerized soluble forms of tmTNF should mimic receptor activation via tmTNF [168,368]. Several TNFR2 agonists have already been developed such as the TNFR2-specific variant of mouse TNF that is trimerized using the trimerization domain of chicken tenascin C as has been done in TNCscTNF80. This drug protected against graft-versus-host disease (GVHD) via host T_reg_ expansion [369]. Lamontain and colleagues recently confirmed that stimulation with TNCscTNF80 effectively leads to expansion of T_regs_ and ameliorates established CIA in mice [370]. Another TNFR2-selective TNF mutein EHD2-scTNFR2 consists of a covalently stabilized human TNFR2-selective single-chain TNF fused to the dimerization domain EHD2 derived from the heavy chain domain of IgEs [174]. TNFR2 agonists could rescue human neurons from death induced by oxidative stress or stimulate T_regs_ in type I diabetes. These could be useful in patients with TNF-mediated neurodegeneration or as a correctional therapy in diabetes patients [371]. Additionally, TNFR2 is expressed by all diseased CD8^+^ T cells and TNFR2 agonism has been shown to selectively kill insulin-autoreactive CD8^+^ T cells in blood of diabetic patients by an altered signaling pathway [184]. This approach is very attractive and is preclinically assessed for type I diabetes and other autoimmune diseases. However, despite the promising features of TNFR2 agonism, it can also be a risky approach as it might enhance the accumulation of pathogenic T cells [372]. In type I diabetes, it has been suggested that tmTNF signaling via TNFR2 is responsible for islet destruction, arguing for TNFR2 antagonism instead [373]. Interestingly, Dong et al. combined TNFR1 antagonism and TNFR2 agonism in a model of *N-*methyl-d-aspartate (NMDA)-induced acute neurodegeneration. Administration of ATROSAB or of a TNFR2-selective TNF mutant EHD2-scTNFRR2 reverted neurodegeneration-associated memory impairment and protected cholinergic neurons against cell death [174]. In addition of the use of TNFR2-agonizing TNF muteins, one may also consider co-administration of the cholesterol lowering drug lovastatin. Indeed, in further support of the importance to preserve TNFR2 signaling in AD, it was shown that statins reduced progression of AD in clinical trials or even prevented the onset of it [374,375], and lovastatin established this by increasing TNFR2 expression. Additionally, the drug protected primary cortical neurons against glutamate-induced excitotoxicity [376].

In contrast to TNFR2 agonism, TNFR2 inhibition was proposed as an effective anti-cancer strategy as TNFR2 has been identified as a human cancer oncogene. Indeed, many cancer cells are characterized by TNFR2 expression that promotes the expansion of tumor cells [14]. Recently, TNFR2 has also been found on the surface of a highly immuno-suppressive tumor-infiltrating subset of T_regs_ [109], and therapies that target and eliminate T_regs_ are currently investigated as cancer treatment [377]. TNFR2 antagonism could therefore act as a double-edged sword as the group of Faustman elegantly showed. TNFR2 antibodies directly blocked the tumor growth, inhibited T_reg_ proliferation and enabled T effector cell expansion which could help amplify effective anti-tumor immune responses [15]. Furthermore, the combination of TNFR2 antagonists with immunotherapeutic stimulants synergistically improves the therapeutic efficacy in colon cancer mouse models [378].

### 6.3. TNF-Inducing Vaccines

Interestingly, also vaccines that stimulate endogenous TNF release are currently evaluated as a long-term modulating immuno-intervention in clinical trials in type 1 diabetes and have been investigated in MS (NCT02081326; NCT00607230; NCT00202410) [349,379]. These approaches are less toxic than recombinant TNF treatment, and can be established via immunization with the Bacillus Calmette-Guérin (BCG) vaccine. This live tuberculosis vaccine contains *Mycobacterium bovis*, known to stimulate the innate immune system by inducing the host to produce TNF that subsequently kills the autoreactive T cells. BCG vaccination of longstanding type I diabetic subjects led to more death insulin-autoreactive T cells and transiently induced beneficial T_regs_ for 4–6 weeks after vaccination [379]. This vaccine is now evaluated in a phase II trial with a duration of five years to establish the long term consequences [349]. In addition, in MS, the clinical benefit was shown, consistent with the outcome in diabetic patients [380]. Vaccinated RRMS patients had a reduction in disease activity and the progression of brain lesions was prevented. The effects of BCG vaccination were also assessed in subjects with clinically isolated syndromes, and at the end of the five-year trial, 58% of the subjects did not progress into MS whereas this was only 30% of the placebo-treated group, and no adverse events were reported [381].

### 6.4. Selective Targeting of sTNF

Instead of targeting the receptors, approaches that selectively target sTNF have also been proposed. One of the most promising drugs is the dominant negative peptide Xpro1595 [382,383]. Xpro1595 selectively binds to soluble TNF monomers without interfering with tmTNF and forms inactive heterotrimers that are unable to interact with the TNFR [382]. Interestingly, XPro1595 suppressed inflammation in both the CIA and the mouse collagen antibody-induced arthritis (CAIA) model for RA without compromising the innate immunity to *L. monocytogenes* infection [382]. The efficacy of XPro1595 has also been tested in other preclinical models. Indeed, in two mouse MS model, EAE and the cuprizone model, subcutaneous injection of XPro1595 was therapeutic and promoted axon preservation and remyelination [164,165,167] and in AD 5xFAD mice it decreased Aβ plaque load and rescued impaired long-term potentiation [189]. Locomotor functioning was improved after spinal cord injury, but only after central administration [163] and intraocular administration of XPro1595 promoted retinal ganglion cell survival in a rat model of ocular hypertension glaucoma [384]. Icv infusion of XPro1595 also prevented the development of depressive-like symptoms induced by exposure to artificial light at night [385]. Peripheral administration of XENP345, a PEGylated variant of XPro1595, in the 6-OHDA model for PD attenuated nigral and dopaminergic cell loss [196,198] (cf. Section 3.4.4). Finally, in the established mouse model of Huntington’s disease (R6/2 mice), particularly icv injection of XPro1595 improved the functional outcome of these mice [386]. Vaccination with virus-like particles of the bacteriophage Qbeta that was covalently linked to sTNF led to Abs specifically neutralizing sTNF. These endogenously raised Abs protected mice from inflammation in RA mouse models [274].

### 6.5. Cell-Type Restricted TNF(R) Targeting

Cell-type restricted targeting of TNF is a new innovative approach that has been suggested by the group of Nedospasov [387]. TNF signaling represents a complex network, and the deleterious or beneficial outcome of TNF depends not only on the receptor via which it signals, but also on the physiological circumstances and the cell type. Indeed, cell-type specific conditional TNF knockout mice point to differential roles for myeloid and T cell-derived TNF in host defense and in several inflammatory mouse models (Table 3). For the immune response against *L. monocytogenes*, both myeloid and T cell-derived TNF have a similar contribution depending on the bacterial load [387]. The situation in different during *M. tuberculosis* infection in which T cell-derived TNF is needed to control the infection and can better not be blocked, whereas myeloid cell-derived TNF is dispensable [40]. For the formation of GCs, another source of TNF, namely B cells, is important. However, to maintain the formation of the GCs and FDC networks in lymph nodes, B cell-TNF synergizes with T cell-TNF [388]. In disease settings, TNF derived from myeloid cells was generally found to have detrimental functions in LPS/d-galactosamine induced hepatotoxicity, in experimental arthritis and in EAE [147]. Conditional ablation of macrophage TNF led to protection against diabetic nephropathy in streptozotocin-induced diabetes [389]. Conversely, T cell-derived TNF demonstrated nonredundant protective roles in these diseases, although in EAE also pathogenic effects of T cell-derived TNF are described [147]. Interestingly, intestinal inflammation could be induced by chronic TNF expression by the IECs whereas in the T cell transfer model of murine colitis, TNF from non-T cells seems to be responsible for colitis induction [390]. Intestinal pathology in TNF^∆ARE^ mice is induced by TNF derived from innate (myeloid cells) and adaptive (CD8^+^ T cells) effector cells as well as IECs via interacting with TNFR1 on mesenchymal cells [85,391,392]. These examples led to the idea of selective inhibition of TNF produced by myeloid cells thereby sparing the protective effects of TNF that is produced by other cells such as T cells [393]. To establish site-directed TNF neutralization myeloid cell-specific TNF inhibitors (MYSTIs) were designed that locally enrich at the cell membrane. These VHH-based bispecifics consist of one arm that binds TNF and another arm directed against the myeloid surface markers F4/80 or CD11b. These drugs retain endogenously generated TNF in vitro, and in vivo the F4/80-directed MYSTI protects against LPS/D-Gal lethal toxicity and was also active in the anti-collagen antibody transfer arthritis model [394,395]. Additionally, treatment with these drugs led to beneficial outcomes in an in vivo model of acute hepatotoxicity as macrophage-derived TNF is directly captured at the source of production [394,396].

Interestingly, in the TNF-induced shock model IEC-restricted TNFR1 expression is sufficient to induce IEC-apoptosis, but chronic targeting of IECs by endogenous TNF is not enough to induce IBD pathology indicating that TNFR1 expressed on other cell types is required for effective pathology [68,69,397]. This means that selective IEC targeting of TNFR1 is not interesting to consider as IBD therapy. In TNF^∆ARE^ mice, the RA pathology is independent of TNF-mediated adaptive immune responses (T and B cells), but it is mediated by TNFR1 on joint (synovial) fibroblasts [85,391,398]. In addition, astrocyte-specific TNFR1 blockage might be considered to prevent memory deficits in MS [172] or CD8 T cell-TNFR2 antagonism for induction of apoptosis in autoreactive lymphocytes in diabetes [184].

Instead of targeted TNF neutralization, there are also several indications that might benefit from targeted TNF supply, for example to exploit its anti-cancer properties. Unfortunately, TNF’s intrinsic and unacceptable toxicity hampers its use as immunotherapeutic. This obstacle can be circumvented by the creation of Activity-on-Target cytokines (AcTakines). These immunocytokines consist of mutated cytokines with reduced binding affinity coupled to a targeting moiety that guides the cytokine to the desired cell target. The activity of the mutated cytokine is only restored after local enrichment at the targeted cell types. This strategy will greatly reduce the off-target adverse effects and improve the desired efficacy [399].

### 6.6. Multispecific Approaches

Simultaneous blockage of multiple pathways might directly cope possible compensation mechanisms that neutralize the initial effect of the drug. Alternatively, this approach can also directly increase the potency of drugs. In the pathogenesis of psoriasis, a prominent role for TNF has been described and this is illustrated by the success of the TNF inhibitors in this disease. In addition to TNF, recent data also suggest an important role for type I IFN in psoriasis. Interestingly, blockage of TNFR1 or the IFN receptor 1 (IFNAR1) only partially protects mice against imiquimod-induced psoriasis, whereas double KO mice lacking both receptors showed superior protection in this model. This was explained by the presence of a sustained type I IFN production in TNFR1 single KO mice [324]. As there is also a clear synergy between TNF and IL17A or IL17F, bispecific antibodies that bind both TNF and IL-17 were designed with the intend to be superior for the treatment of RA, PsA and AS, and to overcome limited therapeutic responses obtained with single cytokine neutralization [401]. Two different bispecific biologics (e.g., COVA322 and ABT-122) are currently under clinical investigation, but the clinical development of COVA322 was terminated due to safety issues (NCT022437870). ABT-122 is a dual-variable domain IgG under clinical development by AbbVie and seems promising after evaluation in several phase I and II trials in healthy volunteers and in subjects with RA and PsA [402,403]. Similar approaches were inquired with the TNF/IL-6 and TNF/IL-23 neutralizing Abs that were patented [404,405]. The first approach could ameliorate the clinical progress in CIA more than the single treatments did [406]. Additionally, RA patients were treated with the combination of IL1 and TNF neutralizing drugs but this therapy did not have added value [407], although it seemed promising in preclinical sepsis models and in acute myeloid leukemia [79,408]. However, the complete blockage of host defense mechanisms should be kept in mind as a plausible destructive side effect. In the context of sepsis, our group developed a bispecific Nb that targets MMP8 and TNFR1, as discussed in Section 3.2.2. In addition to these double cytokine-hitters, the combination between anti-TNF with anti-angiogenic agents were studied in the context of RA. The bispecific Zybody that was generated by the genetic fusion of Ang2-targeting peptides to the heavy chain of an anti-TNF Ab showed superior efficacy compared to the single hit strategy [409].

The establishment of a bispecific therapeutic also allows targeting of cytokine-neutralization on cytokine-producing cells (cf. Section 6.5) or at particular anatomical sites such as regions of inflammation. For instance, anti-TNF Nbs that are coupled to anti-albumin do not only have improved pharmacokinetic properties, but also accumulate in the inflamed joints of CIA mice [410]. Alternatively, in an attempt for improved local delivery to the inflamed joint, TNF-inhibitors were coupled to a single-chain variable fragment (scFv) that recognize collagen type II that is post-translationally modified by reactive oxygen species (ROS) [411], and the so-called MYSTIs introduced in the previous section, also aim for site-directed TNF neutralization. Others also investigated the possibility of simultaneous targeting of MMP14 or cadherin-11 together with TNF in an attempt to direct the pannus-cartilage junction in RA-affected joints [393].

## 7. Concluding Remarks

It is clear from the increasing amount of studies regarding the differential roles of the TNF receptors, the TNF format, i.e., tmTNF or sTNF, and the function of TNF derived from specific cell subsets, that the use of anti-TNF drugs can be improved or adjusted to the specific disease context. Given the relatively long-time experience with the approved anti-TNF drugs, they can be considered as relatively safe; however, improvements are warranted. In addition to the disease settings for which a market authorization is settled, many other diseases in which a specific TNF subtype/receptor is involved can be approached. The examples are numerous and exceed the field of neurology although this was mainly the focus in this review. Selective TNFR1 targeting is described for dry-skin induced chronic itching [412], neuropathic pain [136], ventilator-induced acute lung injury [356], eye disorders [203], and cardiomyopathy and myocardial ischemic injury [413,414,415,416], and this list is not limited to these examples.

Although the expression of TNFR2 is limited in healthy conditions, in (autoimmune) diseases, the expression of TNFR2 is significantly elevated. Importantly, TNFR2 has a superior toxicity profile because of its limited tissue expression compared to the ubiquitous expression of TNFR1 [349]. As discussed in the manuscript, TNFR2 agonism is an attractive checkpoint therapy to modulate the immune regulation through T_reg_ activation, which can be very interesting in, for instance, graft-versus-host disease [369]. Therapies that combine the two strategies in diseases, in which TNFR1 signaling leads to devastating outcomes and stimulation of TNFR2 is needed to promote proliferation or regeneration, to achieve replenishment of the nonfunctional T_reg_ population are attractive.

Additionally, as discussed, cell-specific drugs that are directed against the intended TNFR expressed by a specific cell type are interesting for future research. This would lead to local accumulation, less undesired side effects and thus safer therapies. Collectively, during the development of new innovative therapies, one can not only consider the removal of the detrimental cells or signals that are present, e.g., blockage of the inflammatory TNFR1 pathway or removal of autoreactive T cells, but also think about therapies that stimulate the cells or cellular properties that are defective such as suppressive cell types. Therefore, therapeutic treatments should always find the ideal balance between doing enough good and preventing bad.

In addition, critical notes are needed, and considerable attention should be paid to adverse effects that might pop-up with TNFR1 antagonizing treatments, as the sensitivity to several infectious diseases is clearly increased in mice lacking TNFR1 [417]. However, cell-specific TNFR1 targeting might again offer a solution to increase the safety. Besides, antagonizing a receptor always carries the risk that the receptor is not blocked but instead becomes activated. This event has already previously been described by others, and even led to early termination of a clinical trial [358]. Seemingly, TNFR1 antagonists could not only be converted into potent receptor activators by the induction of TNFR1-oligomerization, but agonistic activities can also be induced upon cross-linking by secondary Abs such as by drug-induced antibodies or pre-existing antibodies that cluster the drug/receptor complex and consequently activate the downstream pathway [358,418,419,420].

To conclude, therapeutic manipulation of TNF remains a very attractive field, and although we know already a lot about the biology of TNF, there is a lot to uncover. This will allow us to select the appropriate treatment for a specific patient population. In addition, improved patient empowerment will drive the development of innovative medicines that deliver more relevant and impactful patient outcomes [421]. Indeed, precision and personalized medicine are currently booming and the needs of the patients should be considered when developing new drugs. Therefore, not only a profound molecular understanding of the considered diseases is indispensable, but also the challenges faced by the patients during their everyday living and their QoL should be accounted for during drug development. Hence, a collaborative approach is essential and will facilitate the introduction of real personalized medicine into clinical practice.

## Figures and Tables

**Table 1:** Evidence for specific TNF(R) targeting in neuronal disease.

Model	In Vivo/In Vitro	Mechanism	References
**Multiple Sclerosis**			
TNFR1/TNFR2^−/−^, TNFR1^−/−^, TNFR2^−/−^ in EAE	In vivo	TNFR1^−/−^ mice are resistant to disease development	[160,161,171]
		TNFR2^−/−^ mice display exacerbated disease progression	
TNF^−/−^ and TNFR1^−/−^ mice in EAE	In vivo	TNF protects against EAE chronicity by inducing regression of myelin-reactive T cells, independent of TNFR1	[96]
TNF^∆1–12^ mice in EAE	In vivo	tmTNF protects against disease development	[162]
TNFR2^−/−^ in EAE and bone-marrow transplantations	In vivo	TNFR2 on non-hematopoietic cells is required for T_reg_ functioning	[181]
OLG-specific TNFR2 KOs (CNP-cre TNFR2^fl/fl^) in EAE	In vivo	OLG-TNFR2 drives OPC differentiation	[177]
CX3cr1-cre TNFR2^fl/fl^ and LysM-cre TNFR2^fl/fl^ in EAE	In vivo	TNFR2 ablation in microglia leads to early EAE onset	[173]
		TNFR2 ablation in monocytes results in EAE suppression	
TNFR1^−/−^ that conditionally re-express TNFR1 in astrocytes in EAE (hGFAPcreT2/tnfr1^cneo/cneo^)	In vivo	Astrocyte TNFR1 mediates learning memory impairment	[172]
TNF^−/−^, TNFR1^−/−^ and TNFR2^−/−^ in CPZ model	In vivo	TNFR2 is critical for OLG proliferation and remyelination	[33]
TNFR2^−/−^ in CPZ model	In vivo	Astrocyte TNFR2 mediates OPC proliferation and differentiation via CXCL12	[179]
CNS-overexpressing TNF mice in TNFR1 or TNFR2 KO background	In vivo	TNFR1 induces OLG apoptosis	[157]
Neuron or astrocyte-overexpressing tmTNF mice	In vivo	Astrocyte tmTNF but not neuron-specific TNF triggers CNS inflammation and neurodegeneration	[145]
TNFR1 inhibition in EAE	In vivo	Disease development is reduced	[169,170]
Nanobody-based TNFR1 inhibition in EAE	In vivo	Prophylactic and therapeutic administration prevents or stops disease development	[171]
sTNF inhibition in EAE	In vivo	Functional outcome is improved	[165]
sTNF inhibition in EAE and in astrocyte-neuron coculture	In vivo	tmTNF is neuroprotective via NF-κB	[164]
	In vitro	sTNF inhibition protects against glucose deprivation	
sTNF inhibition in CPZ model	In vivo	tmTNF is neuroprotective and is needed to maintain myelin sTNF inhibits remyelination and repair	[167]
Astrocytes-OPC co-culture	In vitro	Astrocyte TNFR2 promotes OLG maturation via LIF	[178]
Rat microglia and OLG	In vitro	tmTNF kills OLGs more efficiently than sTNF	[146]
Alzheimer’s Disease			
5xFAD/Tg197 mice	In vivo	Peripheral hTNF mediates reduced amyloidosis and more microglial and astrocytic activation, but also synaptic loss	[185]
TNFR1-overexpressing primary neurons and TNFR1^−/−^ neurons	In vitro	TNFR1 mediates Aβ-induced neuronal death	[129]
APP/PS1 TNFR1^−/−^ and icv AβO injection in TNFR1^−/−^	In vivo	TNFR1 mediates AD-mediated choroid plexus inflammation and TNFR1^+/+^ mice are protected against cognitive decline, microgliosis and amyloidosis	[186]
APP23 TNFR1^−/−^	In vivo	TNFR1 signaling enhances BACE1 activity and Aβ production. Absence of TNFR1 leads to less memory deficits, neuronal loss and microglia activation	[131]
APP23 TNFR2^−/−^	In vivo	Exacerbated AD pathology	[187]
Nanobody-based TNFR1 inhibition icv AβO injection	In vivo	TNFR1 inhibition prevents against cognitive decline	[186]
sTNF inhibition in 3×Tg-AD mice	In vivo	sTNF inhibition reduces APP accumulation in hippocampus and restores synaptic dysfunction	[188,189]
TNFR2 inhibition in SH-SY5Y cells	In vitro	Enhances Aβ toxicity	[190]
Neuronal TNFR2 knockdown in 3×Tg-AD mice	In vivo	Enhances Aβ and Tau-pathology	[191]
Transverse hippocampus slices of WT or TNFR1^−/−^ mice	In vitro	TNF via TNFR1 is a critical mediator of the Aβ-induced inhibition of LTP	[192]
Parkinson’s Disease			
Double TNFR^−/−^ mice in MPTP model	In vivo	Mice were protected against neurotoxicity, but hippocampal vulnerability increased	[193,194]
TNFR1^−/−^, TNFR2^−/−^ mice in MPTP model	In vivo	Neither TNFR1 nor TNFR2 KO mice were protected against MPTP neurotoxicity	[195]
sTNF neutralization in 6-OHDA model	In vivo	Reduced nigral dopaminergic loss and microglia activation	[196,197,198]
Spinal Cord Injury and Traumatic Brain Injury			
sTNF inhibition in SCI	In vivo	Protective	[163]
Double TNFR^−/−^ mice subjected to TBI	In vivo	Bigger lesion volume and BBB impairment	[199]
TNFR1^−/−^ and TNFR2^−/−^ subjected to controlled cortical impact brain injury	In vivo	TNFR1 exacerbates cognitive functioning, TNFR2 attenuates it	[200]
TNFR1^−/−^ and TNFR2^−/−^ subjected to TBI	In vivo	TNFR1 exacerbates neurobehavioral deficits and tissue damage, TNFR2 is protective	[201]
Stroke, Ischemia and Oxidative Stress			
Double TNFR^−/−^ mice in stroke model	In vivo	More neuronal damage and less injury-induced microglial activation	[138]
TNFR1^−/−^, TNFR2^−/−^ and double TNFR mice in focal cerebral ischemia/reperfusion	In vivo	TNFR1 limits neuronal damage and prevents hippocampal degeneration	[202]
TNFR1^−/−^ and TNFR2^−/−^ in model retinal ischemia	In vivo	TNFR1 augments neuronal death, TNFR2 promotes neuroprotection via PI3K-PKB/Akt pathway	[203]
TNFR2 agonist in LUHMES cells treated with H_2_O_2_	In vitro	TNFR2 promotes anti-apoptotic response via PI3K-PKB/Akt pathway	[168]
hTNFR2-expressing OLG + TNFR2 agonist treated with H_2_O_2_	In vitro	TNFR2 protects OPC against oxidative stress	[175]
Excitotoxicity and Seizures			
TNFR2 agonism or TNFR1 inhibition in NMDA-induced neurodegeneration	In vivo	TNFR1 inhibition/TNFR2 agonism protects cholinergic neurons against cell death and reverts neurodegeneration-associated memory impairment	[174]
TNFR1^−/−^ and TNFR2^−/−^ mice on kainate seizures	In vivo	TNFR2 exerts anticonvulsant effects, TNFR1 mediates excitotoxicity	[204]
Primary cortical cells treated with glutamate	In vitro	TNFR2 protects neurons against excitotoxic insults via activation NMDA-receptor	[128]
Microiontophoretic administration of glutamate to TNFR1^−/−^ or TNFR2^−/−^ mice	In vitro	TNFR2 protects hippocampal neurons against excitotoxicity	[205]
Brain Inflammation			
Hippocampal TNFR1^−/−^ and TNFR2^−/−^ neurons	In vitro	TNF vulnerability of TNFR2^−/−^ hippocampal neurons is higher than of TNFR1^−/−^ neurons	[130]
Cultured microglia	In vitro	TNFR2 upregulation after inflammatory stimuli and TNFR2-mediated induction of anti-inflammatory pathways	[182]
Neuropathic Pain and Hippocampal Neurogenesis			
Healthy or diseased TNFR1^−/−^ and TNFR2^−/−^ mice	In vivo	TNFR1 is a suppressor of adult neurogenesis, absence of TNFR2 leads to reduced hippocampal neurodegeneration	[132,133,134,135]
TNFR1^−/−^ and TNFR2^−/−^ mice subjected to CCI	In vivo	TNFR1 induces a neuropathic-pain induced depression	[136]
Double TNFR mice and TNFR1 and TNFR2 inhibitors following CCI	In vivo	TNFR1 inhibition prolongs Wallerian degeneration and TNFR1 regulates macrophage influx	[206,207]
		TNFR1 mediates thermal hyperalgesia	

AβO: oligomerized amyloid beta; AD: Alzheimer’s disease; APP/PS1: amyloid precursor protein/presenilin 1; BACE1: beta-secretase 1; CCI: chronic constriction injury; CNP: 2′,3′-cyclic nucleotide 3′-phosphodiesterase; CPZ: cuprizone; CXCR3: CXC motif chemokine receptor 3; EAE: experimental autoimmune encephalomyelitis; icv: intracerebroventricular; KO: knockout; LIF: leukemia inhibiting factor; LTP: long term potentiation; LUHMES: Lund human mesencephalic; LysM: lysin-motif; NMDA: *N*-methyl-d-aspartate; MPTP: 1-methyl-4-phenyl-1,2,3,6-tetrahydropyridine; OLG: oligodendrocytes; OPC: oligodendrocyte precursor cells; PI3K-PKB/Akt: phosphoinositide-3-kinase–protein kinase B/Akt; SCI: spinal cord injury; sTNF: soluble TNF; TBI: traumatic brain injury; Tg: transgenic; tmTNF: transmembrane TNF.

**Table 2:** Anti-TNF biologics that are approved, in the pipeline or discontinued.

**Current Approved Anti-TNF Biologics**					
**Drug**	**Biosimilars**	**Structure**	**Approved Indication**	**Administration Route**	**Ref.**
Infliximab	CT-P13; SB2	Chimeric mAb	RA, PA, psoriasis, AS, UC, CD, pediatric RA & CD	IV, every 8 weeks following loading at week 0, 2 and 6	[253,254,255,256]
Etanercept	GP2015; SB4	Fusion protein: human TNFR2:IgG1-Fc	RA, PA, psoriasis, AS, JIA	SC, every week or every two weeks	[256,257,258]
Adalimumab	ABP501; ZRC3197	Human IgG1 mAb	RA, PA, psoriasis, AS, JIA, CD, hidradenitis suppurativa, uveitis	SC, every two weeks	[256,259]
Certolizumab pegol	NA	Humanized PEGylated Fab’ fragment of humanized iGG1	RA, PA, AS, CD (only in US and Switzerland)	SC, every two weeks	[260]
Golimumab	NA	Human IgG1 mAb	RA, PA, AS, ulcerative colitis	SC, monthly	[261]
**Anti-TNF Biologics in the Pipeline or Discontinued**					
**Drug**	**Biosimilar**	**Structure**	**Disease Indication**	**Clinical Phase/State**	**Ref.**
Infliximab	BOW015; PF-06438179	Chimeric mAb	RA	Phase III/ongoing	[256]
Etanercept	CHS-0214; HD203	Fusion protein: human TNFR2:IgG1-Fc	RA, psoriasis	Phase III/ongoing	[256]
Adalimumab	BI695501; CHS-1420; GP-2017; M923; SB5; ZRC-3197; FKB327	Human IgG1 mAb	RA, psoriasis, AS	Phase I, II and III/ongoing	[256]
Golimumab	ONS-3035	Human IgG1 mAb	RA, PsA, AS, UC	Preclinical	
SSS-07	NA	Humanized mAb	RA	Phase I	NCT02460393
AVX-470; Aveximab-TNF	NA	Polyclonal bovine anti-TNF Ab	UC, NEC	Phase I	[262,263], NCT01759056
CDP571	NA	Humanized IgG4 anti-human TNF mAb	CD	Phase II/discontinued	[264]
Ozoralizumab	NA	Trivalent, bispecific anti-TNF Nanobody	RA, discontinued for AS, CD and PsA	Phase IIa (Japan)/ongoing	[265]
VHH#1-3	NA	Bivalent Nanobody	RA	Preclinical	[266]
Onercept	NA	PEGylated dimeric soluble human TNFR1	CD, psoriasis, PsA, RA, sepsis, endometriosis	Phase II and III/discontinued	[267,268]
Lenercept	NA	Fusion protein: soluble TNFR1:IgG1-Fc	Severe sepsis, septic shock, RA and MS	Phase II/discontinued	[53,153]
Hitanercept (T0001)	NA	TNFR2-Fc fusion protein	RA	Phase I (China)	[269]
HL036	NA	TNFR1 fragment	Dry eye disease	Phase II	NCT03334539
CytoFab	NA	Polyclonal anti-TNF Fab fragment	Severe sepsis, septic shock	Phase II/discontinued	[270]
Pegsunercept	NA	PEGylated sTNFR1	RA	Phase II/discontinued	[271]
TNF-kinoid	NA	Vaccine to induce anti-TNF Ab, recombinant human TNF coupled to carrier protein KLH	CD, RA	Phase II/suspended	[272,273]
CYT007-TNFQb	NA	Vaccine to induce anti-TNF Ab, recombinant TNF coupled to virus-like particles of the bacteriophage Qbeta	Psoriasis	Phase I and II/discontinued	[274]
TfRMAb-TNFR	NA	Fusion protein: extracellular TNFR2 coupled to mAb against TfR	PD, AD, ischemic stroke	Preclinical	[223,242,275]

(m)Ab: (monoclonal) antibody; AD: Alzheimer’s disease; AS: Ankylosing spondylitis; CD: Crohn’s disease; Fab: fragment antigen binding; JIA: juvenile idiopathic arthritis; KLH: keyhole limpet hemocyanin; MS: multiple sclerosis; NEC: necrotizing enterocolitis; PEG: polyethylene glycol; PsA: psoriatic arthritis; PD: Parkinson’s disease; RA: rheumatoid arthritis; TfR: Transferrin receptor; TNFR: TNF receptor; UC: ulcerative colitis; VHH: variable domain of heavy-chain only antibodies NA: non-applicable.

**Table 3:** Distinct functions of TNF produced by T cells and myeloid cells in several experimental mouse diseases.

Disease Model	Cellular Source of TNF		Ref.
	Myeloid Cells	T Cells	
T cell transfer colitis model	Pathogenic	Non-redundant	[390]
TNF^∆ARE^ intestinal inflammation	Pathogenic	Pathogenic	[392]
TNF^∆ARE^ joint inflammation	NA	Non-redundant	[85]
*L. monocytogenes* infection	Protective	Protective	[400]
*M. tuberculosis* infection	Dispensable ^1^	Protective	[40]
Systemic LPS/D-Gal hepatotoxicity	Pathogenic	Dispensable	[40]
Autoimmune arthritis	Pathogenic	Protective	[393]
EAE	Pathogenic during early phase	Protective and pathogenic	[147]
	protective in late phase		
ConA-hepatitis	Pathogenic	Pathogenic	[40]
Diabetic nephropathy	Pathogenic	ND	[389]

^1^ Mediates immune functions of cells at early stages of infections, but dispensable for protection. ConA: Concanavalin-A; EAE: Experimental autoimmune encephalomyelitis; ND: Not determined.

## Abbreviations

6-OHDA	6-hydroxydopamine
ADA	Anti-drug antibody
ADAM	A disintegrin and metalloproteinase
ADCC	Antibody-dependent cellular cytotoxicity
ALS	Amyotrophic lateral sclerosis
AMPA	α-amino-3-hydroxy-5-methyl-4-isoxazolepropionic acid
APC	Antigen-presenting cells
AS	Ankylosing spondylitis
Aβ(O)	(oligomerized) Amyloid beta
BACE1	Beta-secretase 1
BBB	Blood–brain barrier
BCG	Bacillus Calmette-Guérin
CAIA	Collagen antibody-induced arthritis
cAMP	Cyclic AMP
CASP	Colon ascendens stent peritonitis
CD	Crohn’s disease
CDC	Cell-dependent cytotoxicity
CIA	Collagen-induced arthritis
CLP	Cecal ligation and puncture
CNS	Central nervous system
COX-2	Cyclooxygenase-2
CSF	Cerebrospinal fluid
DC	Dendritic cell
DD	Death domain
DILE	Drug-induced lupus erythematosus
DSS	Dextran sodium sulfate
EAE	Experimental autoimmune encephalomyelitis
EMA	European Medicine Agency
FADD	Fas-associated death domain
FcγR	Fcγ-receptor
FDA	Food and Drug Administration
FDC	Follicular dendritic cell
GC	Germinal center
GVHD	Graft-versus-host disease
HT	5-hydroxytryptamine
hTNF	Human TNF
ip	Intraperitoneal
IBD	Inflammatory bowel disease
icv	Intracerebroventricular
IEC	Intestinal epithelial cells
IFN	Interferon
IFNAR	Interferon-α receptor
Ig	Immunoglobulin
IL	Interleukin
JIA	Juvenile idiopathic arthritis
KO	Knockout
LPS	Lipopolysaccharide
LTD	Long-term depression
LTP	Long-term potentiation
LT-α	Lymphotoxin-α
mAb	Monoclonal antibody
MDSC	Myeloid-derived suppressor cells
MMP	Matrix metalloproteinase
MOF	Multiple organ failure
MPTP	1-methyl-4-phenyl-1,2,3,6-tetrahydropyridine
MS	Multiple sclerosis
MTX	Methotrexate
Nb	Nanobody
NEC	Necrotizing enterocolitis
NK	Natural killer
NOD	Non-obese diabetic
OLG	Oligodendrocytes
OPC	Oligodendrocyte precursor cell
PD	Parkinson’s disease
PEG	Polyethylene glycol
PLAD	Pre-ligand assembly domain
PPMS	Primary progressive MS
PsA	Psoriatic arthritis
QoL	Quality-of-life
RA	Rheumatoid arthritis
ROS	Reactive oxygen species
RRMS	Relapsing-remitting MS
scFv	Single-chain variable fragment
SIRS	Systemic inflammatory response syndrome
SLE	Systemic lupus erythematosus
sTNF(R)	Soluble TNF(R)
TACE	TNF-α converting enzyme
TDM	Therapeutic drug monitoring
TIMP	Tissue inhibitor of metalloproteinase
tmTNF	Transmembrane TNF
TNF	Tumor necrosis factor
TNFR	Tumor necrosis factor receptor
TRADD	TNFR1-associated death domain
TRAF(2)	TNF receptor-associated factor (2)
TRAPS	TNF receptor-associated periodic syndrome
T_regs_	Regulatory T cells
UC	Ulcerative colitis
VHH	Variable domain of heavy-chain only Abs
VNAR	Variable new antigen receptor
WT	Wild type
